# Metagenomic community composition and resistome analysis in a full-scale cold climate wastewater treatment plant

**DOI:** 10.1186/s40793-022-00398-1

**Published:** 2022-01-15

**Authors:** Paul Jankowski, Jaydon Gan, Tri Le, Michaela McKennitt, Audrey Garcia, Kadir Yanaç, Qiuyan Yuan, Miguel Uyaguari-Diaz

**Affiliations:** 1 Department of Microbiology, University of Manitoba, 45 Chancellors Circle, Buller Building, Winnipeg, MB R3T 2N2 Canada; 2 Department of Medical Microbiology and Infectious Diseases, University of Manitoba, Winnipeg, MB Canada; 3 Clayton H. Riddell Faculty of Environment, Earth, and Resources, University of Manitoba, Winnipeg, MB Canada; 4 Department of Civil Engineering, University of Manitoba, Winnipeg, MB Canada; 5 Present Address: Institute of the Environment, University of Ottawa, Ottawa, ON Canada

**Keywords:** Antibiotic resistance genes, Wastewater treatment, Metagenomics, Resistome, Integrases, Bacterial community, Bacteriophages

## Abstract

**Background:**

Wastewater treatment plants are an essential part of maintaining the health and safety of the general public. However, they are also an anthropogenic source of antibiotic resistance genes. In this study, we characterized the resistome, the distribution of classes 1–3 integron-integrase genes (*intI1, intI2, and intI3*) as mobile genetic element biomarkers, and the bacterial and phage community compositions in the North End Sewage Treatment Plant in Winnipeg, Manitoba. Samples were collected from raw sewage, returned activated sludge, final effluent, and dewatered sludge. A total of 28 bacterial and viral metagenomes were sequenced over two seasons, fall and winter. Integron-integrase genes, the 16S rRNA gene, and the coliform beta-glucuronidase gene were also quantified during this time period.

**Results:**

Bacterial classes observed above 1% relative abundance in all treatments were Actinobacteria (39.24% ± 0.25%), Beta-proteobacteria (23.99% ± 0.16%), Gamma-proteobacteria (11.06% ± 0.09%), and Alpha-proteobacteria (9.18 ± 0.04%). Families within the Caudovirales order: *Siphoviridae* (48.69% ± 0.10%), *Podoviridae* (23.99% ± 0.07%), and *Myoviridae* (19.94% ± 0.09%) were the dominant phage observed throughout the NESTP. The most abundant bacterial genera (in terms of average percent relative abundance) in influent, returned activated sludge, final effluent, and sludge, respectively, includes *Mycobacterium* (37.4%, 18.3%, 46.1%, and 7.7%), *Acidovorax* (8.9%, 10.8%, 5.4%, and 1.3%), and *Polaromonas* (2.5%, 3.3%, 1.4%, and 0.4%). The most abundant class of antibiotic resistance in bacterial samples was tetracycline resistance (17.86% ± 0.03%) followed by peptide antibiotics (14.24% ± 0.03%), and macrolides (10.63% ± 0.02%). Similarly, the phage samples contained a higher prevalence of macrolide (30.12% ± 0.30%), peptide antibiotic (10.78% ± 0.13%), and tetracycline (8.69% ± 0.11%) resistance. In addition, *intI1* was the most abundant integron-integrase gene throughout treatment (1.14 × 10^4^ gene copies/mL) followed by *intI3* (4.97 × 10^3^ gene copies/mL) while *intI2* abundance remained low (6.4 × 10^1^ gene copies/mL).

**Conclusions:**

Wastewater treatment successfully reduced the abundance of bacteria, DNA phage and antibiotic resistance genes although many antibiotic resistance genes remained in effluent and biosolids. The presence of integron-integrase genes throughout treatment and in effluent suggests that antibiotic resistance genes could be actively disseminating resistance between both environmental and pathogenic bacteria.

**Supplementary Information:**

The online version contains supplementary material available at 10.1186/s40793-022-00398-1.

## Background

Wastewater treatment plants (WWTPs) are essential to maintain quality of life by protecting public health and aquatic organisms. They serve as a centralized system that collects wastewater for large-scale treatment to reduce the contamination of downstream aquatic environments. Wastewater treatment consists of removing nutrients, solids, and microbial biomass, which is critical in reducing the impact of releasing wastewater into the environment [1, 2]. However, these water infrastructures were not designed to efficiently remove all chemical and biological pollutants. Specifically, WWTPs have been shown to contain diverse communities of environmental and pathogenic bacteria as well as antibiotics, pharmaceutical products, and heavy metals that remain throughout the treatment processes [2–4]. Although chemical pollutants in wastewater such as antibiotics and their metabolites are found in lower and potentially subinhibitory concentrations ranging from 0.1 to 1.4 ppb [5–7], their presence is concerning given their potential role as a selective pressure for the exchange of resistance genes [3, 8].

The large diversity and abundance of bacteria within wastewater combined with selective pressures from antibiotics and disinfectants creates a hotspot for the development and transfer of antibiotic resistance between bacteria [9, 10]. Mobile genetic elements (MGE) such as plasmids, transposons, and integrons are well characterized genetic elements important for the transfer of antibiotic resistance genes (ARGs) by conjugation and transformation [8, 10]. Phages, viruses that infect bacteria, have recently gained attention as another source of ARGs that can transfer resistance through transduction [11, 12]. Phages are the most abundant biological entity within any given environment with an estimated abundance of 10^31^ viruses worldwide [13, 14]. We are only beginning to characterize and recognize the importance of this diversity, not only as a source of new genes, but also as a pool of ARGs accessible by bacteria [11, 12, 15]. As a major link between society and the environment, WWTPs receive sewage water from many sources including households, hospitals, and industry. This represents an ideal environment for the transfer of ARGs between bacteria and from phages to bacteria. This poses concerns about the accumulation of antibiotic resistance and the potential to transfer ARGs to clinically relevant pathogens that may be present within wastewater [16]. As antibiotic resistance accumulates both clinically and in the environment, we continue to descend towards a post-antibiotic era with antibiotics becoming less effective and viable as a therapeutic option for treating bacterial infections [16, 17]. To prevent this loss of antibiotic effectiveness, it is imperative that we mitigate the spread of antibiotic resistance by reducing opportunities for horizontal gene transfer [18]. These strategies include both clinical and environmental considerations to curtail ARG dissemination. Recent advancements along with the reduction in cost of high-throughput culture-independent methods such as next-generation sequencing have improved our ability to monitor ARG dissemination [19–21].

In the present study, we use metagenomic shotgun sequencing and real-time PCR to characterize the composition of both bacteria and phage diversity as well as the resistome of major treatment processes within the North End Sewage Treatment Plant (NESTP) located in Winnipeg, Manitoba, Canada during the fall and winter months. This study serves as a baseline for the continued monitoring of ARG dissemination over each season, which will contribute to our understanding of antibiotic resistance and community structures within various wastewater treatment processes. This information is essential for future large-scale upgrades of the NESTP targeting phosphorus and nitrogen removal as well as microbiological changes occurring within WWTPs. This will enable us to monitor the effects of modifying operational parameters on microbial diversity and the resistome as well as introduce modifications to the process aimed at reducing the release of ARGs and antibiotic resistant bacteria into the aquatic environment.

## Materials and methods

### WWTP processing and sample collection

The NESTP is the largest wastewater facility in the Province of Manitoba (49°57′08.1″N 97°06′11.4″W) operating year-round with seasonal temperatures ranging from − 40 to 30 °C with the current study ranging from − 18.6 to 2.8 °C. This WWTP serves roughly 70% of the population in the city of Winnipeg treating an average of 200 million liters per day. Treatment begins with the removal of large solids followed by smaller solids and oils in primary clarification. Wastewater is then moved to high purity oxygen bioreactors for biological treatment where it is inoculated with activated sludge returned from secondary clarification to remove nutrients, biosolids, and other organic compounds. This is followed by secondary clarification which removes the remaining small solids through sedimentation before the wastewater is subjected to UV disinfection. The total hydraulic retention time of wastewater in the NESTP is 12 h. The removed biosolid waste is subjected to anaerobic sludge digestion for 25 days followed by dewatering for disposal. UV-treated final effluent water quality parameters are summarized in Table 1. Influent and effluent water quality datasets are included in Additional file 2. Raw sewage or untreated influent (RS), returned activated sludge (RAS), and UV-treated final effluent (EFF) were collected on the following dates from the NESTP: October 22nd, 2019 (T1), November 28th, 2019 (T2), December 18th, 2019 (T3), and February 6th, 2020 (T4). Dewatered sludge (SC) was also collected during the T3 and T4 sampling events. One liter of each sample was collected in sterile containers and transported on ice to the laboratory where they were stored at 4 °C. Within 24 h of collection, aqueous treatment samples were filtered through a sterile cheesecloth to remove large solids and 100 to 200 mL of the filtrate was filtered through 0.2-µm 47-mm Supor-200 membrane filters (Pall Corporation, Ann Harbor, MI) to capture bacterial cells for nucleic acid extraction. A filtration control consisting of 200 mL of Milli-Q water was also prepared for each sampling event. Filters were collected and stored at − 20 °C for further processing.

**Table 1:** UV-treated final effluent water quality parameters of the North End Sewage Treatment Plant

	Sampling time				Mean	Standard deviation
	Oct-22-2019 (T1)	Nov-28-2019 (T2)	Dec-18-2019 (T3)	Feb-6-2020 (T4)^‡^		
pH	7.12	6.81	6.79	6.99	6.93	0.14
Turbidity (NTU)	12.25^†^	4.2	6.3	9.2	7.99	3.03
TSS (mg/L)	19.5^†^	6	10	18	13.38	5.58
BOD (mg/L)	19.5^†^	13	18	26	19.13	4.64
COD (mg/L)	51.5^†^	66	94	89	75.13	17.25
TN (mg/L)	15.4^†^	40.7	49.8	50.5	39.1	14.22
TP (mg/L)	1.67^†^	3.43	1.69	1.69	2.12	0.76
TOC (mg/L)	19.8^†^	21.2	29.6	34.4	26.25	6.02
Precipitation (mm)*	4.8	0	1	1.6	1.85	1.8
Grab temperature (°C)	13.4	14.1	14.1	12.7	13.6	0.7
*E. coli* (counts/100 mL)	60	60	90	1080	322.5	437.51

TSS, total suspended solids; BOD, biochemical oxygen demand; COD, chemical oxygen demand; TN, total nitrogen; TP, total phosphorus; TOC, total organic carbon

*Cumulative amount of rainfall over three days

^†^Parameters measured the day before and the day after were averaged and used to estimate parameters of sample date

### Bacterial fraction DNA extraction

For the aqueous treatment samples (RS, RAS, and EFF), the 0.2-µm filters were washed with 15 mL of 1 × PBS-Tween20 solution and homogenized at 2500 rpm for 15 min. Supernatants were then transferred to a fresh tube and centrifuged at 3300×*g* for 15 min to pellet down cells. Pellet was resuspended and transferred to PowerBead tubes for nucleic acid extraction using the DNeasy PowerLyzer PowerSoil Kit (Qiagen Sciences, Maryland, MD) as per the manufacturer’s instructions. For the dewatered sludge samples, ~ 5 g of solids was collected into 30 mL of 1 × PBS-Tween20 solution, vortexed until homogeneous (2500 rpm for 15 min) and centrifuged at 4 °C and 4500×*g* for 20 min. The resulting pellet (0.25 g) was then transferred to a PowerBead tube and extracted using the DNeasy PowerLyzer PowerSoil kit as per the manufacturer’s instructions, while the supernatant was kept for further filtration and DNA extraction of viral particles.

### Viral fraction DNA extraction

The 0.2-µm filter flow-through from the aqueous treatment samples was collected and stored at 4 °C. Dewatered sludge viral DNA extraction followed the bacterial DNA extraction protocol scaled up to 30 g followed by supernatant filtration through 0.2-µm filter repeated twice. For both sets of samples, 140 mL of filtrate was transferred into Centricon Plus-70 filter units (Millipore Corporation, Billerica, MA). Filtrates were concentrated to ~ 250 µL according to the manufacturer’s instructions with additional modifications described here. Each sample was centrifuged at 3000×*g* for 30 min at 20 °C in 70 mL increments. The supernatant was discarded after each run. The Centricon Plus-70 filter units were then inverted, and the concentrated viral fraction was transferred to sterile tubes and centrifuged at 800×*g* for 2 min at 20 °C. The ultrafiltrate was pre-treated with 2U of Turbo DNase and 60 µg of RNAse A (ThermoFisher Scientific, Waltham, MA, USA). Nucleic acid was extracted using the QIAamp MinElute Virus Spin kit (Qiagen Sciences, Maryland, MD) following the manufacturer’s instructions including the Qiagen Protease and omitting the carrier RNA step. Nucleic acid was eluted with 50 µL of AVE elution buffer.

### Mock community

Positive sequencing controls were created for each bacterial and viral sequencing batch. Bacterial mock community consisted of equal amounts of *Escherichia coli* (ATCC 25922), *Salmonella enterica* (ATCC 13076), *Pseudomonas aeruginosa* (ATCC 10145), *Staphylococcus aureus* (ATCC 25923), *Legionella pneumophila* (ATCC 33152), *L. longbeachae*, clinical isolates of *Campylobacter lari* and *C. upsaliensis*, and environmental isolates of *C. jejuni* and *C. coli* with a total concentration of 11.2 ng/µL. The viral mock community consisted of equal amounts of Adenovirus and Myophages M2 and M3 with a total concentration of 0.4 ng/µL. Nucleic acid was extracted from the bacterial mock community using the DNeasy PowerLyzer PowerSoil Kit (Qiagen Sciences, Maryland, MD) according to the manufacturer’s instructions. Viral mock community nucleic acid was extracted using the QIAamp MinElute Virus Spin kit (Qiagen Sciences, Maryland, MD) including the modifications described above (see Methods: “Viral fraction DNA extraction” section).

### DNA precipitation and sequencing

All extracted bacterial DNA samples were precipitated using 0.1 volumes of 3 M sodium acetate (pH 4.6), two volumes of 100% ethanol, and 4 µL of 5 mg/mL linear acrylamide, which was stored at − 80 °C overnight. Samples were centrifuged at 16,000×*g* for 30 min at 4 °C. Supernatants were discarded and pellets were washed with 1 mL of ice-cold 70% ethanol before repeating centrifugation. Resulting pellets were air-dried and resuspended in 50 µL of 10 mM Tris solution. DNA concentration was measured using the Qubit 4.0 fluorometer and Qubit dsDNA High Sensitivity Assay Kit (Invitrogen, Carlsbad, CA). Metagenomic shotgun sequencing was performed on the Illumina NextSeq platform (Illumina, Inc., San Diego, CA) at 1 × depth using the Illumina Nextera Flex kit with 300 bp paired-end outputs at the Integrated Microbiome Resource (IMR, Halifax, NS). Raw sequencing read adaptor sequences were trimmed prior to receiving sequencing reads. Raw metagenomic sequencing reads are available in the NCBI Sequence Read Archive under BioProject ID: 768945.

### Quantification of gene copy numbers and statistical analysis

Real-time PCR TaqMan assays were carried out for each sample (RS, RAS, EFF, and the negative filter control) in triplicate. Mobile integron-integrase genes (classes 1–3) served as biomarkers for measuring variation in ARG dissemination between treatments [8, 22]. Coliform beta-glucuronidase gene *uidA* was used as a culture-independent method of *E. coli* and coliform quantification [23] in addition to plate counts reported in Table 1. The 16S rRNA gene was quantified to assess total bacterial abundance. Sequence and product size of primers and probes are listed in Table 2. Real-time PCR data for the dewatered sludge was unavailable for the present study.

**Table 2:** Description of primers and probes used for quantitative PCR

Gene	Primer name	Sequence (5′–3′)	Product size (bp)	References
*intI1*	IntI1-LC1	GCCTTGATGTTACCCGAGAG		Barraud et al. [22]
	IntI1_LC5	GATCGGTCGAATGCGTGT	196	
	IntI1_probe	(FAM) ATTCCTGGCCGTGGTTCTGGGTTTT (/ZEN, IABkFQ)		
*intI2*	IntI2_LC2	TGCTTTTCCCACCCTTACC		Barraud et al. [22]
	IntI2_LC3	GACGGCTACCCTCTGTTATCTC	195	
	IntI2_probe	FAM-TGGATACTCGCAACCAAGTTATTTTTACGCTG (/ZEN, IABkFQ)		
*intI3*	IntI3_LC1	GCCACCACTTGTTTGAGGA		Barraud et al. [22]
	IntI3_LC2	GGATGTCTGTGCCTGCTTG	138	
	IntI3_probe	(FAM) CGCCACTCATTCGCCACCCA (/ZEN, IABkFQ)		
*uidA*	784F	GTGTGATATCTACCCGCTTCGC		Frahm and Obst [23]
	866R	AGAACGGTTTGTGGTTAATCAGGA	84	
	EC807	(FAM) TCGGCATCCGGTCAGTGGCAGT (/ZEN, IABkFQ)		
16s rRNA gene	Bac1055YF	ATGGYTGTCGTCAGCT		Ritalahti et al. [26]
	Bac1392R	ACGGGCGGTGTGTAC	~ 320 bp	
	Bac1115P	(FAM) CAACGAGCGCAACCC (/ZEN, IABkFQ)		

Each 10 µL TaqMan real-time PCR mixture consisted of 5 µL of TaqMan Environmental Master Mix 2.0 (Applied Biosystems, Foster City, CA), 400 nM of each primer, 100 nM of each probe, and 2 µL of 10 ng/µL template DNA. Real-time PCR was performed using an ABI QuantStudio 5 Real-Time PCR System (Applied Biosystems, Foster City, CA). Thermal cycling conditions were the same for all targeted genes: 2 min incubation at 50 °C, denaturation and activation of *Taq* polymerase for 10 min at 95 °C, followed by 40 cycles of 15 s at 95 °C and 60 s at 60 °C. Primers and probes are listed in Table 2. Primers were used in the Primer-BLAST tool [24] to extract target regions. Then, these regions were uploaded to Geneious Prime R9 [25] to corroborate primer and probe sequences of gene fragments. Combined integron-integrase gene primer sequences and *uidA* primer sequences were used to construct gBlock Gene Fragments (IDT, Coralville, IA). These gBlock constructs were used to generate standard curves for the quantification of environmental gene copy numbers (GCN).

The 16S rRNA gene standard curve was generated with DNA from *Salmonella enterica* (ATCC 13076). GCN of all target genes were calculated and normalized per milliliter of sample filtered and per nanogram of DNA using equations described by Ritalahti et al. [26] and Lee et al. [27]. GCN were log_10_-transformed for generalized linear model (GLM) analysis using Statistical Analysis System (SAS University Edition for Windows). Tukey–Kramer tests were run to determine statistical differences between GCN using GLM results for treatment conditions and across sampling events (T1 through T4) [28]. Differences between conditions were considered statistically significant at a p-value ≤ 0.05.

### Metagenomic data processing and analysis

**Taxonomical characterisation**. Raw metagenomic shotgun sequencing reads were processed in Geneious (version 9.0.5) by pairing and merging paired-end reads before filtering sequence lengths below 151 bp to remove short and low-quality reads [29]. These reads were then used as input for MG-RAST to characterize the community composition through sequence similarity searches using default parameters [30]. Metagenomic sequences are available from MG-RAST with sequence IDs listed in Additional file 1: Table S1. Principal coordinate analysis (PCoA) with Bray–Curtis distance matrix was performed using abundance data at the species taxonomic level using the phyloseq R package [31]. Alpha-diversity indices (Shannon diversity, Simpson diversity, and Chao1) and rarefaction curves were generated using vegan R package [32]. Data visualization was performed using Tableau [33] and ggplot2 [34] in RStudio [35]. Alpha-diversity values were log_10_-transformed for generalized linear model (GLM) analysis and Tukey–Kramer test using SAS 9.4M6 to determine statistical differences between treatments [28].

**ARG analysis**. The merged and filtered paired-end reads were used to generate contigs with the Geneious (version 9.0.5) de novo assembler tool at medium-fast sensitivity using default settings [25, 29]. The contigs (range 151 bp to 197 kb) were then compared against the comprehensive antibiotic resistance database (CARD) using the resistance gene identifier tool (RGI) to identify ARGs for the characterization of the resistome [36]. The ARGs identified by CARD RGI were grouped into classes according to the type of antibiotic they confer resistance against and quantified by absolute abundance as well as relative abundance (number of ARG class divided by total ARG) and normalized abundance (number of ARG class divided by the total number of bacterial and viral reads) reported in percentage.

**Network analysis and visualization**. Network analysis was performed to investigate co-occurrences between ARGs and bacterial and phage families in the different wastewater sample types. Microsoft Excel was used to organize data into a suitable format for R [37]. RStudio [35], was used to generate Spearman’s correlation matrices and the preliminary edge and node lists needed for network visualization. The R packages used are listed in Additional file 1: Table S2. The edge and node lists were finalized using Microsoft Excel. Gephi [38] was then utilized for network visualization. Gephi’s output files were aesthetically enhanced in Inkscape [39].

## Results and discussion

### Taxonomical composition of the North End Sewage Treatment Plant

#### Bacterial community composition

**Fig. 1:**
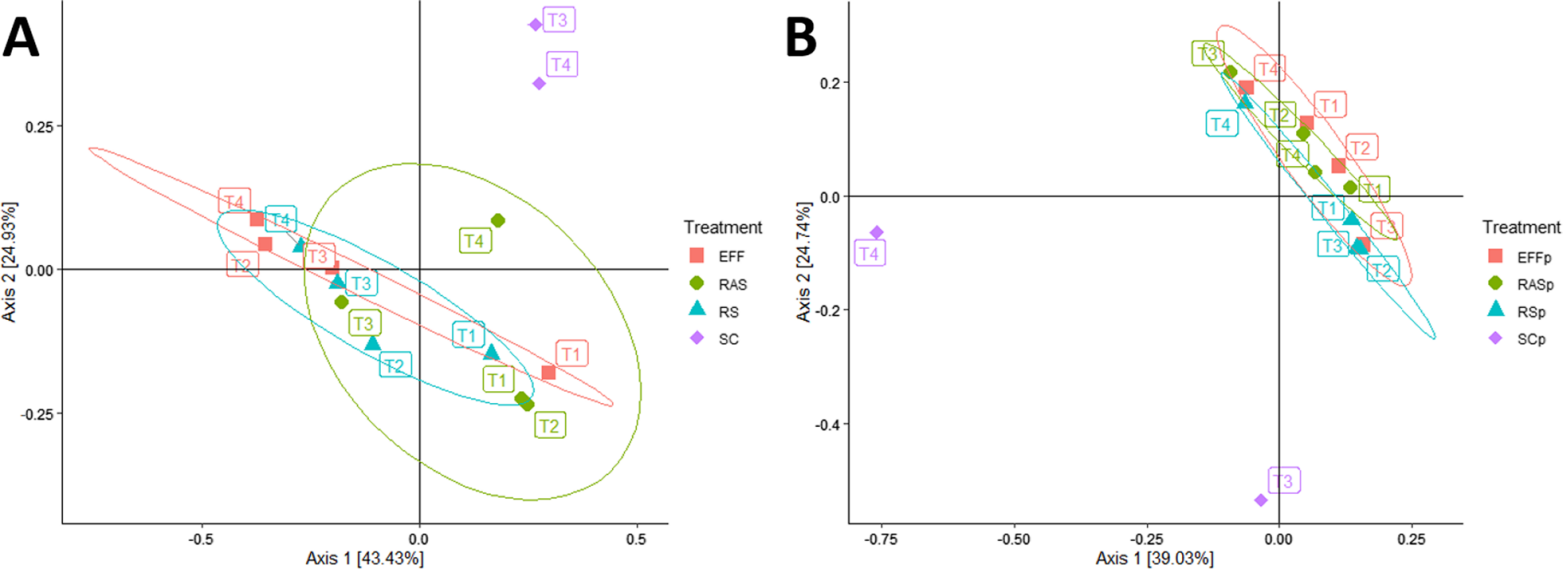
Principal coordinates analysis across treatments using the Bray–Curtis distance matrix at the species level. **A** Comparison of the bacterial samples stratified by domain, principal components 1 and 2 explain 68.36% of the variation. **B** Comparison of the phage samples stratified by domain, principal components 1 and 2 explain 63.77% of the variation. Effluent (EFF), returned activated sludge (RAS), raw sewage/influent (RS), dewatered sludge (SC), negative control (NEG), October (T1), November (T2), December (T3), February (T4), notation of “p” indicates phage sample

**Fig. 2:**
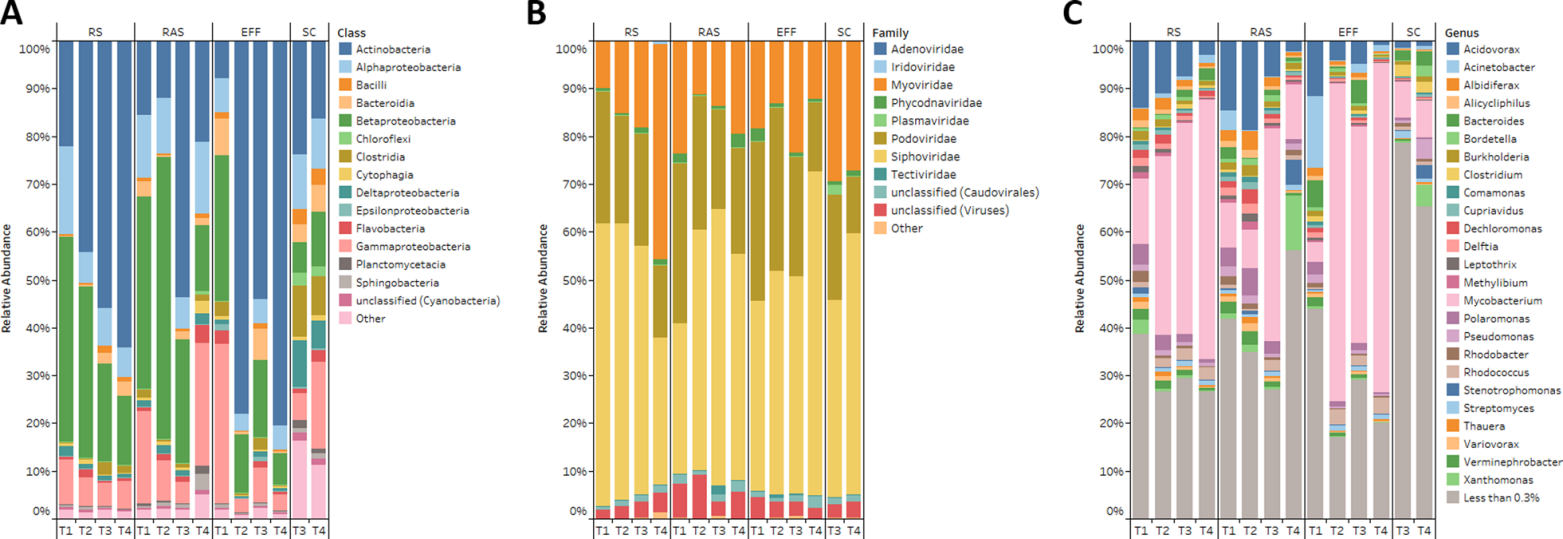
Composition of bacteria and phage in the North End Sewage Treatment Plant. **A** Relative abundance of bacteria at the class level. **B** Relative abundance of phage at the family level. **C** Relative abundance of bacteria at the genus level. Effluent (EFF), returned activated sludge (RAS), raw sewage/influent (RS), negative control (NEG), October (T1), November (T2), December (T3), February (T4)

**Fig. 3:**
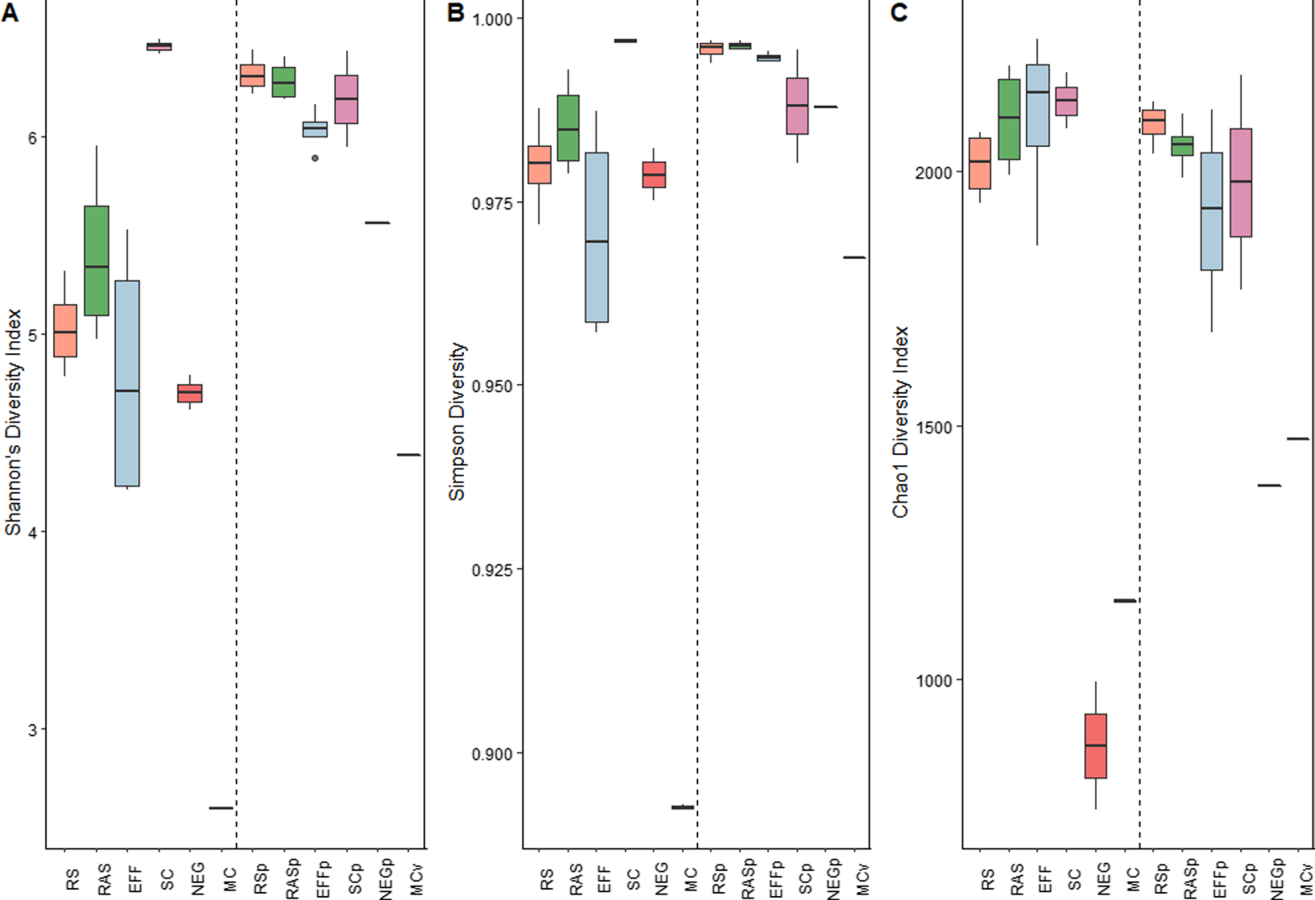
Alpha diversity indices of each treatment at the species level. **A** Shannon’s diversity index. **B** Simpson diversity index. **C** Chao1 index

The major bacterial classes found in almost all samples above 1% relative abundance were Actinobacteria (39.24% ± 0.25%), Betaproteobacteria (23.99% ± 0.16%), Gammaproteobacteria (11.06% ± 0.09%), Alphaproteobacteria (9.18 ± 0.04%), and Bacteroidia (2.63% ± 0.02%). This is consistent with other reports of WWTP class composition [40–42]. PCoA (Fig. 1) and relative abundance comparisons (Fig. 2a) were performed to determine the relatedness of samples and fractions across treatments and sampling events. Bacteria PCoA (Fig. 1a) shows clustering of the RS, RAS, and EFF samples together by treatment and by month. SC samples were more similar to each other but clustered away suggesting compositional differences after anaerobic digestion and the dewatering process. Indeed, SC had significantly higher diversity that was more evenly distributed compared to EFF by Shannon (p = 0.02) and Simpson diversity indices (p = 0.01) (Fig. 3a and b). SC also had higher diversity compared to RS and RAS, but this was not significant. Additionally, the relative abundance of bacterial classes in the SC samples appeared to be more evenly distributed than the other sample types (Fig. 2a). The Chao1 diversity index showed similar richness across treatments with no significant differences detected (Fig. 3c). Each sampling event also displayed differences in clustering with the samples from T1 and T3 clustering closely together while T2 and T4 separated across the x-axis (Fig. 1a). A potential explanation for this grouping is the fluctuation in the relative abundance of Actinobacteria, Beta-proteobacteria, and Gamma-proteobacteria across samples for both T2 and T4 (Fig. 2a). This suggests that there were shifts in the relative dominance of certain classes of bacteria over the course of treatment during these two time points. Curiously, the negative controls for T1 and T2 sampling events had similar alpha-diversity scores for both Shannon and Simpson diversity, yet a lower score for Chao1 was recorded (Fig. 3). Inspection of the genus dataset (see Additional file 2) and relative abundances (Additional file 1: Figure S2a) reveals that most of the represented genera in the negative controls match previously reported genera found in the kitome suggesting contamination related to the DNA extraction kit [43]. The presence of kitome contaminants in T3 and T4 negative controls could not be evaluated due to insufficient number of reads for analysis in MG-RAST (Additional file 1: Table S1).

The bacterial community composition at the genus level for RS, RAS, EFF, and SC for each sampling event is displayed in Fig. 2c. Average percent relative abundance in RS, RAS, EFF, and SC respectively, consisted of, but was not limited to, *Mycobacterium* (37.4%, 18.3%, 46.1%, and 7.7%), *Acidovorax* (8.9%, 10.8%, 5.4%, and 1.3%), *Polaromonas* (2.5%, 3.3%, 1.4%, and 0.4%), *Acinetobacter* (0.9%, 1.2%, 4.6%, and 0.5%), *Xanthomonas* (1.1%, 3.7%, 0.3%, and 2.4%), *Bacteroides* (1.1%, 1.1%, 2.7%, and 2.4%), *Albidiferax* (1.8%, 2.1%, 0.9%, and 0.3%), *Rhodococcus* (2.0%, 1.1%, 2.2%, and 0.9%), *Verminephrobacter* (1.4%, 1.7%, 0.9%, and 0.2%), and *Pseudomonas* (0.9%, 1.4%, 0.8%, and 2.4%). *Nitrospira*, a common genus contributing to nitrite oxidation typically found in high abundance (around 2% to 6%) in WTTPs [44–47] had an average abundance of 0.06% in the NESTP. *Acidovorax, Pseudomonas,* and *Xanthomonas* have previously been reported as being highly abundant in activated sludge as common denitrifying bacteria [48–50]. Interestingly, *Polaromonas*, a psychrophile most abundant on glacier surfaces, was found to have a high relative abundance throughout wastewater treatment [51, 52]. The presence of *Polaromonas* suggests that it could potentially play a role in biochemical cycling in the cold climate found during winter in Manitoba [51, 53]. *Mycobacterium* was the most abundant genus throughout treatment apart from the SC samples where genera were more evenly distributed (Figs. 2c, 3b). This trend was also observed through real-time PCR by Amha et al. where the relative abundance of non-tuberculoid *Mycobacterium* species in wastewater increased after chlorine treatment [54]. These results may be explained by the ability of *Mycobacterium* species to aggregate together in aquatic environments. Bohrerova and Linden demonstrated that increasing aggregate sizes of *M. terrae* were able to better survive UV inactivation compared to non-aggregated samples [55]. The composition of bacteria in RAS was different from several reports of activated sludge composition sharing only a few genera in common [44–46, 48, 49, 51]. The bacterial mock community provides support for the presence of the genera observed as all of the spiked-in species were represented at the genus level with high relative abundance (Additional file 1: Figure S2b). Although there was a high amount of richness observed in the mock community (Fig. 3c), this could be explained by possible kitome contamination during extraction [43] as well as the use of a large number of reads and default MG-RAST annotation parameters during annotation resulting in inflated richness [56, 57].

The ESKAPE group (consisting of *Enterococcus faecium, Staphylococcus aureus, Klebsiella pneumoniae, Acinetobacter baumannii, Pseudomonas aeruginosa,* and *Enterobacter* spp. [58]) of nosocomial pathogens are a major issue causing both clinical and economic burden across the world due to their propensity to develop multidrug resistance against virtually all available antimicrobial agents [59]. Although resistance often develops through person-person transmission and misuse of antimicrobials, environments such as wastewater represent an additional milieu for the transfer of MGEs between environmental and pathogenic bacteria [16, 17]. In fact, the ESKAPE group has been detected in effluent [60] and dewatered sludge in the current study. All representatives of this group were detected across all sampling sites and sampling events (see Additional file 2). This suggests that MGEs carrying ARGs may not only be transferred to other bacteria, but to these nosocomial pathogens as well. Although we cannot detect whether ARGs were obtained by this group, their presence in this selective environment is concerning given the potential to acquire resistance. Furthermore, the release of these pathogens in downstream aquatic environments and in dewatered sludge, often used as agricultural fertilizer, poses the risk of reintroducing these pathogens into anthropogenic settings where they can further endanger public health [41, 61].

#### Composition of the NESTP phageome

Taxonomic identification of the DNA phages was completed with MG-RAST and the composition of the phageome is reported at the family taxonomic level in Fig. 2b. The most abundant phages within the NESTP phageome belong to the tailed dsDNA phages within the order Caudovirales. *Siphoviridae* was the most abundant family with a relative abundance of 48.69% ± 0.10% across all samples followed by *Podoviridae* (23.99% ± 0.07%), *Myoviridae* (19.94% ± 0.09%), and unclassified Caudovirales (1.47% ± 0.01%). These phages comprise the majority of DNA phages found in the NESTP (Fig. 2b). This is in accordance with metagenomic studies of phages in wastewater [62–64] as well as in a freshwater ecosystem [65]. Members of the Caudovirales order infect many of the genera present in the NESTP including *Mycobacterium, Acinetobacter, Pseudomonas, Salmonella, Escherichia,* and *Staphylococcus* [63, 66]. Other viruses identified were *Phycodnaviridae* (1.08% ± 0.01%) and *Tectiviridae* (0.27% ± 0.01%). Neither of these low abundance viruses were detected in the negative control suggesting that they were likely present within the NESTP (Additional file 1: Figure S2c; see Additional file 2). Additionally, the taxonomic assignment of the spiked-in virus families in the viral mock community were correctly identified at the family and genus level (see Additional file 2; Additional file 1: Figure S2d), lending further support for the accurate detection of viral families by MG-RAST. Although at the genus level, several other marine bacteria were also identified in addition to the *Synechococcus* DC2 strain used to culture *Myophage* M2 and M3 suggesting possible bacterial contamination during the isolation of phage from seawater [67]. The presence of *Phycodnaviridae*, a large dsDNA virus that infects eukaryotic algal cells [68], in the wastewater samples suggests that phytoplankton and other algal cells may also be present during wastewater treatment. Previous metagenomic studies have reported *Phycodnaviridae* as a lesser abundant virus in wastewater [64, 69].

Further investigation of the phage metagenomes by the alpha-diversity indices in Fig. 3 show that these metagenomes were highly diverse and evenly dispersed. Linear regression analysis of the changes in diversity and evenness across treatments was not significant suggesting that treatment did not affect phage diversity (Fig. 3). Each phage metagenome was also compared by PCoA (Fig. 1b) revealing clustering of RS, RAS, and EFF samples together indicating similarity across treatments. The T3 and T4 SC samples did not cluster together, deviating across the y- and x-axis respectively. When examining the rarefaction curves, phage diversity was not fully captured as most phage metagenomes did not reach an asymptotic plateau (Additional file 1: Figure S1). This suggests that the effect of treatment on lower abundant phage species may not have been adequately detected. This is however expected and consistent with the fact that the majority of phages remain as unidentified dark matter requiring deeper sequencing and improved bioinformatic techniques to capture this diversity [15, 70]. Furthermore, PCoA including all metagenomic sequencing samples demonstrated two completely separated clusters for bacteria and phage (Additional file 1: Figure S3). Alongside the increased percentage of viral reads in the phage samples (4.69–20.01%) compared to bacterial samples (0.03–0.23%) based on MG-RAST annotation (Additional file 1: Table S1), these results indicate that the study methods were effective in reducing bacterial abundance and differentiating the bacterial and phage fractions.

### Comparison of GCN across treatments and time

**Fig. 4:**
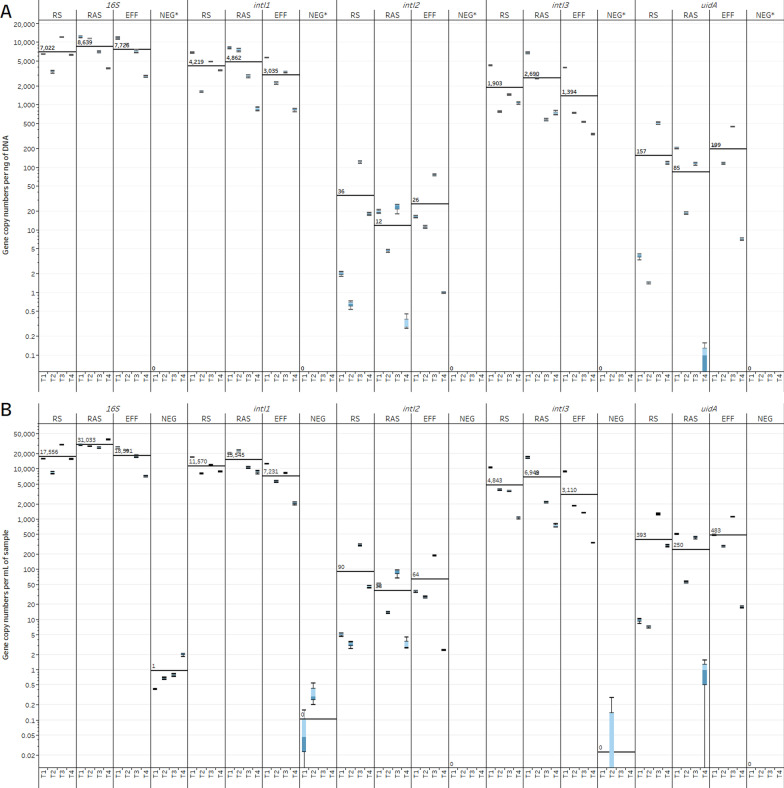
Gene copy numbers of selected genes per milliliter of sample (**A**) and per nanogram of DNA (**B**) for the various wastewater treatment processes over a four-month period. The black lines represent the mean for each treatment over four months. Effluent (EFF), returned activated sludge (RAS), raw sewage/influent (RS), negative control (NEG), October (T1), November (T2), December (T3), February (T4). *DNA concentration was below the detectable limit therefore gene copy number per nanogram of DNA could not be quantified

Integron-integrase MGE biomarkers *intI1*, *intI2*, and *intI3* were quantified as a proxy for measuring variation in ARG dissemination between treatments due to their presence on transposons often associated with conjugative plasmids containing ARGs [8, 18, 71]. Normalized GCN per milliliter and per nanogram of DNA show that *intI1* and *intI3* were the most prevalent integron-integrase genes within all treatments over the four-month period (Fig. 4). However, *intI2* remained at low abundance throughout the treatment with a mean normalized GCN of 6.40 × 10^1^ gene copies per milliliter of sample and 2.50 × 10^1^ gene copies per nanogram of DNA (Fig. 4). Class I integrons carrying *intI1* have previously been shown to be the most abundant class of integrons in WWTPs and often contain clinically important ARGs [9, 18, 72]. Concordantly, *intI1* was the most abundant integrase detected in all treatments with a mean of 1.14 × 10^4^ gene copies per mL and 4.00 × 10^3^ gene copies per ng DNA (Fig. 4). The presence of *intI1* is associated with specific resistance gene families such as streptomycin resistance genes (*aadA*), sulfonamide resistance genes (*sul1* and *sul2*), as well as captured beta-lactam resistance genes [18, 72–74]. The findings of low GCN abundance of *intI2* across the different treatments (Fig. 4) is surprising given its association with common human gut bacteria within the *Enterobacteriaceae* family [28, 74]. Class III integron-integrase *intI3* also had a higher abundance in the NESTP with a mean of 4.97 × 10^3^ gene copies per mL and 1.99 × 10^3^ gene copies per ng DNA. The class III integron-integrases have previously been associated with beta-lactamases *bla*GES and *bla*IMP which were found in several samples in the current study (see Additional file 3), but whether these genes are present within the integron is uncertain [9, 18, 74].

Overall, NESTP wastewater treatment across all measured time points did not significantly change any of the MGE biomarkers targeted in this study (p-value > 0.05). This suggests that stable ARG dissemination within the NESTP may be possible throughout treatment and in the downstream aquatic environment as MGEs were present in UV-treated EFF. All pairwise comparisons between treatments and the negative control for each integron-integrase gene were significant (p-value < 0.05). Pairwise comparison of *intI3* between T1 and T4 across treatments was significantly different (p-value of 0.0074) indicating a decrease in *intI3* GCN towards the colder winter months in Manitoba (Fig. 4) although whether this constitutes seasonal variation or not requires further sampling. No other significant differences were detected between months across treatments.

The 16S rRNA GCN Tukey–Kramer test did not reveal any significant differences between 16S rRNA GCNs across treatments or between treatments across the four time points (p-value > 0.05). Only pairwise comparisons between treatments and the negative control for each month was significant (p-value < 0.0001). These results indicate that there was little variation in bacterial abundance across both treatments and monthly time points. Bacterial abundance measured in GCN per ng of DNA remained consistent through treatment but decreased nearly twofold from RAS to EFF in GCN per ml of sample although this was not significant (Fig. 4). This suggests that UV disinfection was able to reduce bacterial abundance although not as effectively as other studies that demonstrate 16S rRNA log-fold reductions after secondary clarification and UV disinfection [75] as well as chlorine disinfection [20]. The cold temperatures during the sampling events (ranging from -18.6 °C to 2.8 °C) and grab temperatures around 13.6 °C (Table 1) may have reduced the efficacy of UV disinfection although also reducing potential reactivation of UV irradiated cells [76, 77]. Other factors that may have contributed to reducing the effectiveness of UV disinfection include high BOD, COD, TSS, low hydraulic retention time, and precipitation (Table 1) [78]. However, when comparing by bacterial richness and evenness there is an observed increase from RS to RAS with the addition of activated sludge which decreases after UV treatment, although this is not significant (p > 0.05; Fig. 3). There was also a non-significant increase in Chao1 richness in EFF (Fig. 3c) possibly due to the bias of the Chao1 diversity index towards low abundance species [21] which likely occurred after UV disinfection. A similar pattern was observed in WWTPs from comparably cold climates [79].

No significant differences were detected between treatments across the four time points for *uidA* (p-value > 0.05). Although not significant, there was a decrease in average GCN from RS to RAS (Additional file 1: Figure S4). The RS, often containing representative microbial communities of the human gut microbiota [80], had a relative abundance of *uidA* to 16S rRNA gene of 1.55%, which decreased to 0.88% with the addition of activated sludge for biological treatment of the influent (Additional file 1: Figure S4). This suggests a possible dilution of *E. coli* and other coliform bacteria in RAS with the addition of a diverse community of bacteria in the sludge. The subsequent increase in its relative abundance from RAS (0.88%) to EFF (2.44%) however, cannot be attributed to the removal of specific bacteria. Culture-dependent *E. coli* and fecal coliform counts show an average of only 3.2 colonies present per milliliter of EFF (Table 1). The *uidA* results suggest that current UV disinfection practices may be insufficient to reduce *E. coli* and coliforms in EFF. However, culture-dependent counts for *E. coli* are within acceptable limits for the NESTP with the exception of T4 (Table 1). This discrepancy may be explained by the indiscriminate detection of DNA from both viable and nonviable cells by qPCR [81] especially since cold temperatures reduce the potential for UV-irradiated cell reactivation [77]. The difference in *uidA* ratios could also be due to the retention time between influent and effluent as well as UV disinfection efficacy.

### Characterization of the North End Sewage Treatment Plant resistome

The presence and identity of ARGs in each treatment and fraction was determined using CARD [36]. ARGs identified with below 50% identity match to the reference were filtered out. Additionally, the datasets generated for each sample were manually curated to remove inferred resistance through variation of targeted structural genes. These variants are structurally resistant to antibiotics including elfamycin, rifamycin, ethambutol, fluoroquinolones, diaminopyrimidine, and other drugs [82]. This also included removing resistance-nodulation-division efflux pumps because their primary function may not be the efflux of antibiotics [83], and this cannot be elucidated in the present study methods. The remaining resistance genes were grouped into major antibiotic classes: aminoglycosides, beta-lactams, chloramphenicols, fluoroquinolones, glycopeptide antibiotics, macrolides, nucleoside antibiotics, peptide antibiotics, sulfonamides, quaternary ammonium compounds (QACs), tetracyclines, multiple (resistance against 3 or more different antibiotic classes), and other antibiotics (residual efflux resistance genes against antibiotics not belonging to the major classes reported). The curated CARD datasets are found in Additional file 3.

#### Resistome of the bacterial fraction

**Fig. 5:**
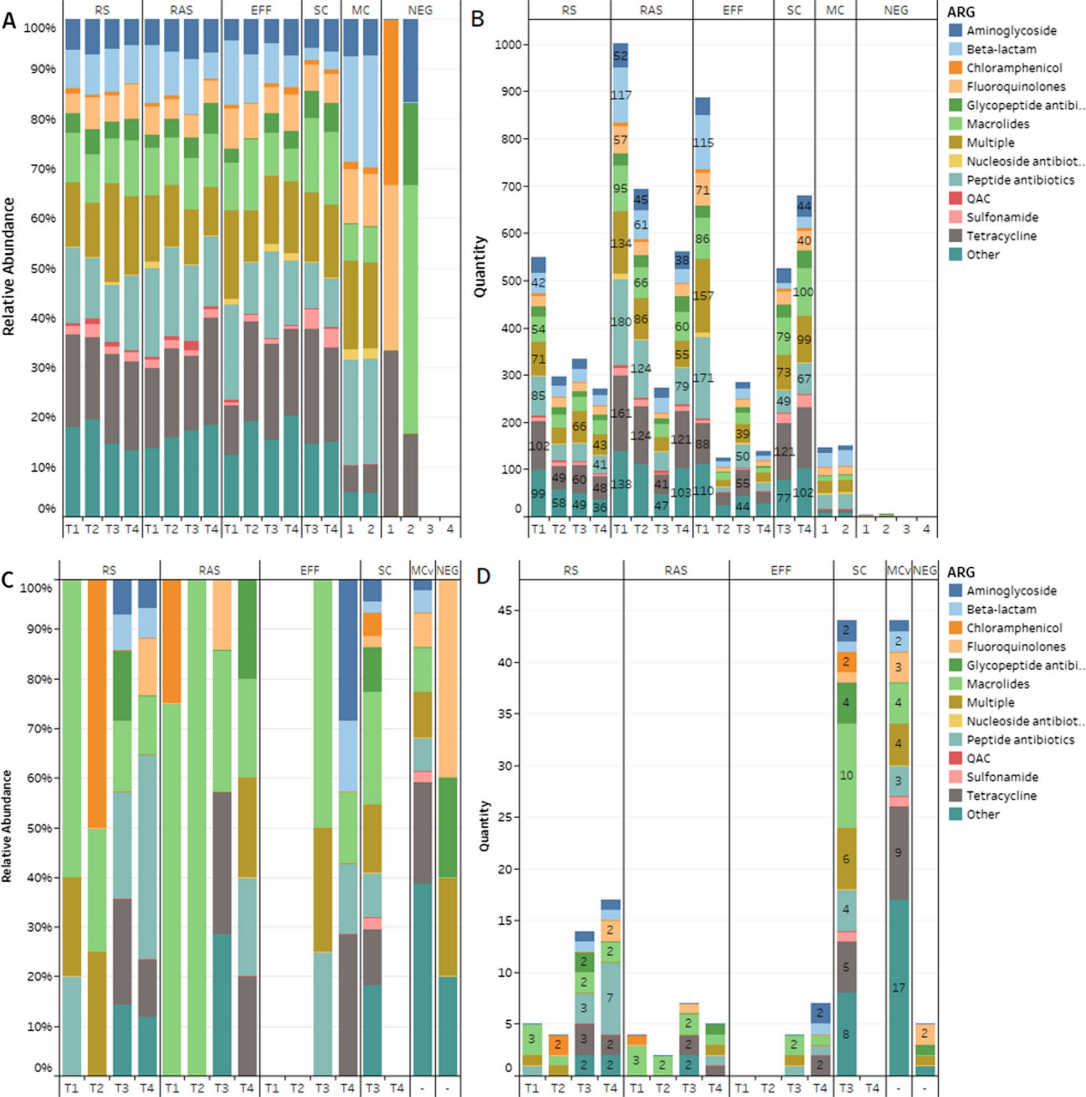
Composition of antibiotic resistance genes in various treatment processes over 4 months. **A** Relative abundance and **B** absolute abundance of antibiotic resistance genes identified by CARD in the bacterial samples. **C** Relative abundance and **D** absolute abundance of antibiotic resistance genes identified by CARD in the phage samples. Effluent (EFF), returned activated sludge (RAS), raw sewage/influent (RS), dewatered sludge (SC), negative control (NEG), October (T1), November (T2), December (T3), February (T4)

Antibiotic resistance genes targeting most classes of antibiotics were detected across wastewater samples. When separated into fractions, the bacterial fractions contained a more diverse set of ARGs across treatments compared to bacteriophages (Fig. 5). Results over the sampling period showed a consistent relative abundance across the different ARG classes in the bacterial fraction (Fig. 5a). The most abundant class of antibiotic resistance in each process was resistance against tetracyclines (17.86% ± 0.03%) followed by peptide antibiotics (14.24% ± 0.03%), macrolides (10.63% ± 0.02%), β-lactams (8.00% ± 0.03%), as well as the other (16.28% ± 0.02%) and multiple (13.63% ± 0.03%) categories (Fig. 5a). Differences in the abundance of ARGs across treatment processes was observed in Fig. 5b where the abundance of ARGs increased from RS to RAS and subsequently decreased in the EFF, except for the T3 sampling event. This trend may well be explained by the introduction of microorganisms within the activated sludge that could carry additional ARGs, contributing to the composition of the resistome. After UV treatment, the number of ARGs decreases with the relative distribution remaining even across ARG classes (Fig. 5a and b). The NESTP reduced the overall abundance of ARGs in the EFF in T2, T3, and T4 (Fig. 5b).

**Fig. 6:**
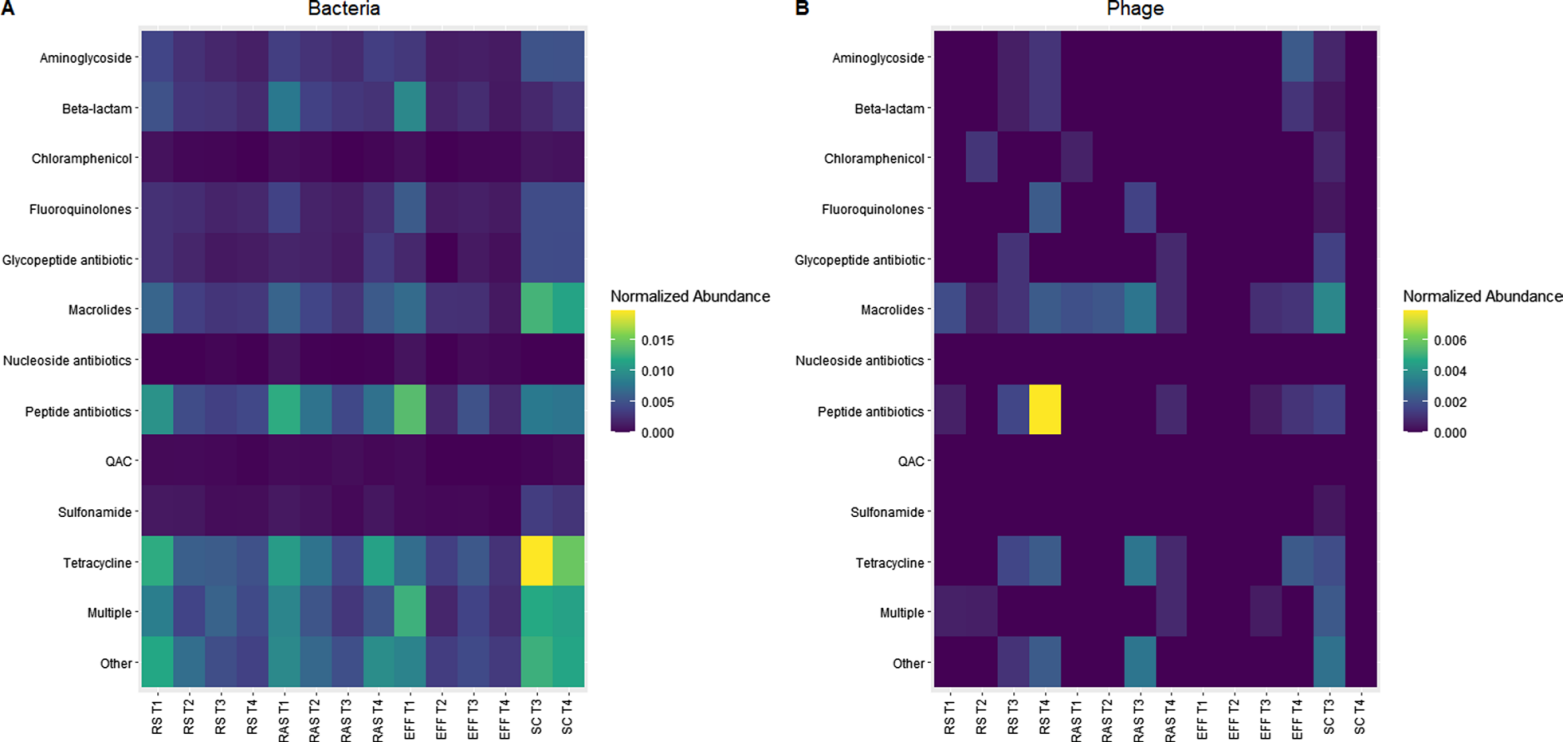
Heatmap of antibiotic resistance classes. **A** Bacterial fraction. **B** Phage fraction. Effluent (EFF), returned activated sludge (RAS), raw sewage/influent (RS), dewatered sludge (SC), October (T1), November (T2), December (T3), February (T4)

The high relative abundance of tetracycline, multiple, and other ARGs was also observed when transformed to normalized abundance although this appears to decrease after UV treatment (Fig. 6a). SC shows an enrichment of ARGs against tetracycline, macrolides, peptide antibiotics, multiple and other efflux mechanisms compared to other T3 and T4 treatments (Fig. 6a). Although this enrichment is not observed to the same degree from RS to EFF, the presence of ARGs in EFF and SC raises concerns about their release and usage for irrigating and fertilizing agricultural soils as this represents a potential source for the reintroduction of antibiotic resistance accumulating pathogens into communities and hospitals [61].

#### Bacterial ARGs of concern

Examination of the curated CARD datasets (see Additional file 3) revealed the presence of many clinically relevant ARGs in the RS, RAS, EFF, and SC. Resistance against vancomycin, a glycopeptide antibiotic used to treat severe gram-positive infections [84], was detected throughout wastewater treatment, with the exception of T2 EFF. Components of the vancomycin resistance operon such as variants of the *vanRS* two component regulatory system were detected alongside the variable ligases *vanA, vanB, vanC, vanD, vanG,* and *vanN* [85]. However, the exact variants present cannot be elucidated at the sequencing depths used. Regardless, the presence of these operons in the WWTP is concerning due to their potential to be located on MGEs capable of transferring to pathogenic gram-positive enterococci or other opportunistic pathogens such as methicillin resistant *Staphylococcus aureus* (MRSA), however, transfer of *vanA*, if present, to MRSA is thought to be rare [85, 86]. Several vancomycin ligases were detected in the EFF and SC. Macrolide resistance was found in all samples and specific mechanisms of macrolide resistance of interest found in wastewater treatment were efflux pumps and ribosomal methyltransferases. Between the EFF and SC samples, MGE-associated efflux and ribosomal methyltransferases resistance genes were detected including *mel, mef, erm(B)*, and *erm(G)* [87]. These resistance genes have been found in several of the ESKAPE group pathogens [87] as well as on transposons associated with *Streptococcus pneumoniae* [88]. The spread of macrolide resistance is important to mitigate as macrolides are one of the most prescribed types of antibiotics in the United States used to treat upper respiratory infections [88]. The presence of other clinically relevant resistance genes such as *bla*_*CTX-M*_, *bla*_*OXA*_, and *bla*_*IMP*_, *mcr* mobile colistin resistance gene variants, and mobile tetracycline resistance genes (*tet(A), tet(B)* and *tet(M)*) were detected in many of the EFF and SC samples (see Additional file 3). Additionally, the *tet(X6)* mobile tigecycline resistance gene was putatively detected in the T1 EFF, T3 SC, and T4 SC. Colistin and tigecycline are last resort antibiotics that were only recently discovered to have mobile resistance genes [89, 90]. The detection of these resistance genes in the NESTP is concerning given their potential for dissemination to clinically relevant pathogens such as those in the ESKAPE group. Overall, the presence of these clinically important ARGs within wastewater at the NESTP raises concerns about the possibility of ARG transfer and increased antibiotic resistance in the environment as a result of these microbial pollutants [16].

#### Resistome of the phage fraction

The phage metagenomes were also run through CARD RGI following the same parameters and filtering criteria as the bacterial fraction. Overall, there was a tenfold decrease in the abundance of ARGs in the phage samples compared to bacteria. This was higher than Wang et al. who reported an overall 18-fold decrease in ARG abundance between fractions [12], although this was in a swine feedlot where the ratio of phage to bacteria may be lower compared to human or other anthropogenic environments [91]. The most common ARGs found in the phage samples were resistance against macrolides (30.12% ± 0.30%), peptide antibiotics (10.78% ± 0.13%), tetracyclines (8.69% ± 0.11%), and multiple (7.40% ± 0.11%) (Fig. 5c). The ARG classes observed for phage are concordant with those found in the bacterial resistome although at differing relative abundances. Overall, the abundance of phage ARGs appears to decrease with each subsequent treatment (Fig. 5d). Discordance in the SC phage ARGs was observed between T3 and T4 with roughly twice the number of ARGs in T3 than any other sample while no ARGs were detected in T4 (Fig. 5d). This suggests that the T3 SC sample may have been contaminated although further monitoring is required to confirm this observation. Macrolides were the most abundant ARGs in the phageome and were consistently observed in RS and RAS before declining in EFF (Fig. 6b). Peptide antibiotics were sparsely distributed across samples with the exception of the T4 RS, which contained several ARGs previously only observed in the bacterial fraction (Fig. 6b). Only two putative β-lactam ARGs were observed in the T3 and T4 RS (Fig. 5d). This result was surprising since multiple studies have reported β-lactam and tetracycline ARGs as the most abundant classes in phage [11, 12, 92]. Nayfach et al. report lincosamides (categorized as macrolides here) as the most abundant ARGs followed by β-lactams and tetracyclines when using CARD RGI [15]. Apart from β-lactams, the ARG profile was similar to that presented here.

#### Detection of clinically relevant ARGs in phage metagenomes

Phage acquisition of ARGs is generally thought to occur through generalized transduction or non-specific packaging of bacterial DNA instead of phage DNA in the capsid [61]. Therefore, bacterial originating ARGs are more likely than *bona fide* phage ARGs. Investigation of the complement of ARGs detected in phage metagenomes revealed a core set of streptogramin resistance genes known as virginiamycin *O*-acetyltransferase (vat) enzymes (see Additional file 3). At least one of the following vat genes were detected in nearly all phage metagenomes: *vatB, vatH, vatI,* and *vatF.* Moon et al. [65] also reported the presence of vat genes (*vatA* and *vatB*) in the phageome of a freshwater river. However, these putative phage associated ARGs were not tested for resistance. The APH(3′)-ia aminoglycoside resistance gene was identified in the T4 EFF with a 94–100% sequence identity match. APH(3′)-ia is a 3′-phosphotransferase that inhibits aminoglycoside antibiotics through the antibiotic target inactivation mechanism [93]. This high confidence match suggests that this ARG may be a true positive although whether this originated from phage or contaminating bacterial DNA is uncertain and warrants further investigation.

#### Considerations and limitations of phage-associated ARG detection

The viral negative control was composed of the flow-through of each negative control generated per sample processing event. A total of roughly 800 mL (200 mL flow-through per event) was collected and concentrated for viral-like particles. This control contained a few ARGs belonging to classes that were less frequently represented in the treatment samples. These consisted of ARGs conferring resistance against fluoroquinolones, glycopeptide antibiotics, multiple, and other residual efflux resistance genes. Only the T3 RAS and SC phage resistome overlapped with the negative control containing the *patB* and *novA* genes. *patB* is an ARG typically associated with *Streptococcus*, which may have been a contaminant during DNA extraction as it has been identified in the kitome [43]. Metagenomic sequencing of the phage samples yielded a similar number of reads to the bacterial samples (Additional file 1: Table S1) with the percentage of phage reads ranging from 4.69% to 20.01% of the total reads based on MG-RAST annotation. This percentage of phage reads in the metagenomes was similar to Moon et al. [65] and higher than that reported by Subirats et al. [11] suggesting that the repeated 0.2 µm filtration of the flow-through before concentrating viral particles was successful in reducing bacterial contamination. The low percentage of phage reads may be partially explained by the misclassification of prophages as originating from bacteria [70]. However, this remains a limitation for the analysis of the phage resistome [11]. A higher input volume to concentrate more virus-like particles may overcome this limitation by increasing the number of phage reads as well as the abundance of ARGs detected. This could also help reduce the risk of false positive ARG calls from potential contaminants. The detection of putative phage ARGs in the current study as well as previous studies [11, 12, 65] suggest that phages may represent an additional reservoir for the propagation of antibiotic resistance through transduction [94]. Future studies should consider increasing metagenomic sequencing depth and using longer reads (e.g., Nanopore technology) to better characterize individual phage. These studies should also aim to isolate phages containing ARGs in order to characterize resistance, host range, as well as transduction rates to determine their potential to spread antibiotic resistance, especially to the ESKAPE group pathogens [94, 95].

### Co-occurrence of bacteria, phage, and ARGs

**Fig. 7:**
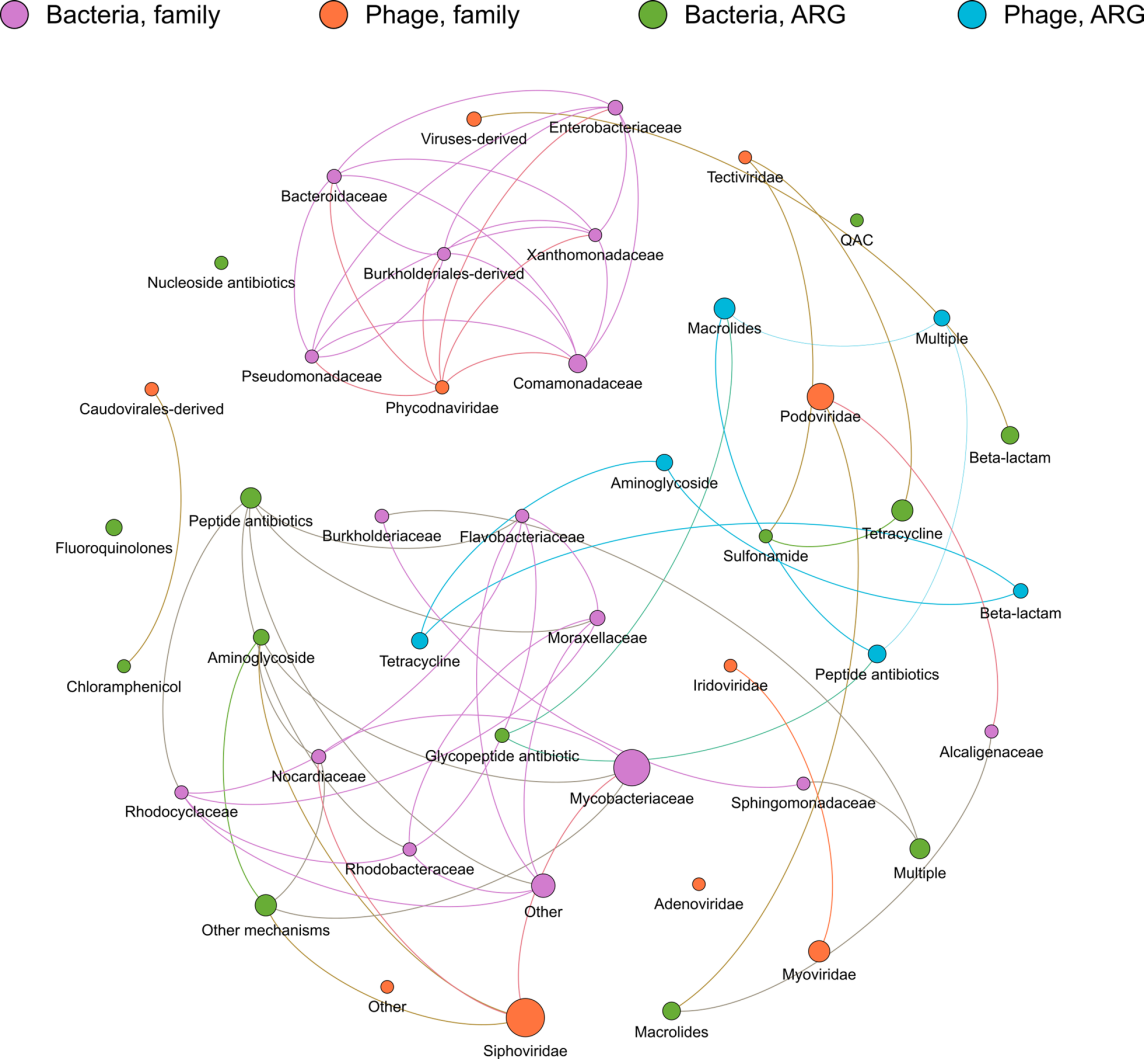
Network analysis of co-occurrence patterns among ARGs and microbial taxa in EFF samples. Node sizes correspond to relative abundances. Antibiotic resistance gene (ARG), effluent (EFF)

We explored co-occurrences between ARGs and bacterial and phage families using network analysis and visualization. Relative abundances were used instead of absolute abundances to eliminate the potential bias that may arise from differences in the number of reads between samples and sequencing runs. Furthermore, only correlations with rho > 0.8 and p-value < 0.01 were included for visualization purposes. Figure 7 illustrates the network generated using information from EFF samples. Corresponding graphs for RS (Additional file 1: Figure S5), RAS (Additional file 1: Figure S6), and SC (Additional file 1: Figure S7) can be found in Additional files.

Figure 7 comprises 45 nodes and 66 edges. The average degree is 2.933, the average path length is 1.015, and the network diameter is 2. The phage families with the highest relative abundances (indicated by the biggest size of their nodes) were *Siphoviridae* and *Podoviridae*. This prevalence is consistent with previous literature [64, 96].

Some of the co-occurrences seen in Fig. 7 may be partially explained by host-phage relationships. For instance, *Siphoviridae* has been reported to infect *Mycobacteriaceae* [97] and *Nocardiaceae* (*Rhodococcus*) [98]. Other potential explanations for co-occurrences involve diet and habitat. For example, *Phycodnaviridae* is a common inhabitant of the human gut [99], which may explain its co-occurrence with several bacterial families, such as *Bacteroidaceae*, *Enterobacteriaceae,* and *Pseudomonadaceae* [91, 100]. Meanwhile, co-occurrences of bacterial ARGs with bacterial families may be partially due to the latter carrying the former. This hypothesis is similar to that posited by Li et al. [101], which suggests that certain microbial taxa carry specific ARGs. Furthermore, phages from shed gut bacteria readily transduce other environmental bacteria, disseminating ARGs in the process [62, 91, 94, 102, 103].

Of the network analyses, the graph for SC samples (Additional file 1: Figure S7) is notable. This network exhibits very strong co-occurrence patterns among all ARGs and phylogenetic groups, which may be explained by the fact that microorganisms and other solids are more densely packed in the SC [104]. These results may suggest that there is an increased exchange of ARGs in the SC compared to other processes [105]. More steps should be taken to further treat this SC since it is used to fertilize farmlands [106] and thus could spread ARGs and pathogens to agricultural products.

In general, the presence of various ARGs and bacterial & phage families in EFF samples indicates that they were not removed by the wastewater treatment process. Thus, discharging EFF into rivers incurs the risk of disseminating pathogens, antibiotic resistance genes, and possibly antibiotic-resistant pathogens. Therefore, future studies should monitor the presence of ARGs as well as bacterial and phage taxa downstream of wastewater treatment plants. Additionally, more studies on the co-occurrence of ARGs and human bacterial pathogens in environmental reservoirs, as well as subsequent analyses, should be conducted to establish and validate newfound potential relationships between ARGs and microorganisms of public health importance.

## Conclusion

The bacterial composition at the class level for each wastewater treatment process closely resembled other studies with a core set of bacteria: Actinobacteria, Betaproteobacteria, and Gammaproteobacteria [40–42]. Further analysis at the genus level revealed that *Mycobacteria*, *Polaromonas*, and *Acidovorax* were found at higher relative abundances with the latter two genera possibly explained by the colder operating temperatures of the NESTP during the Fall and Winter months.

DNA phage in the NESTP consisted almost entirely of *Siphoviridae, Podoviridae*, and *Myoviridae* belonging to the Caudovirales order. These phages are known to infect several of the most abundant bacteria in the NESTP including *Mycobacteria*, *Acinetobacter*, and *Pseudomonas* [63, 66]. Another interesting phage detected at 1% relative abundance was *Phycodnaviridae*, an algae-infecting phage and an inhabitant of the human gut. This phage was found to co-occur with several gut bacteria suggesting that its presence in wastewater is due to human waste rather than from the surrounding environment.

The NESTP resistome consisted of all classes of ARGs studied. The most abundant ARG classes in the bacterial and phage resistomes were resistance against tetracyclines, peptide antibiotics, and macrolides. In the bacterial fraction, several clinically relevant ARGs were detected in the EFF and SC including MGE-associated macrolide ARGs and mobile resistance genes (*mcr* and *tet(X6)*). The phage resistome also contained ARGs largely consisting of streptogramin resistance as well as the aminoglycoside resistance gene APH(3’)-ia identified with high sequence identity match. These results support recent reports of phages carrying ARGs and their potential role in disseminating resistance through transduction [11, 12, 15, 61].

Wastewater treatment effectively reduced the abundance of ARGs from the RS to the EFF in both bacterial and phage samples although certain clinically relevant ARGs remained in both the EFF and SC. This raises concerns about the potential risk of antibiotic resistance dissemination into downstream watersheds and agricultural lands that may use the anaerobically digested biosolids as fertilizer. This dissemination of ARGs downstream of the NESTP may serve as a potential route for reintroduction into communities and healthcare settings.

Further analysis of integron-integrase MGE biomarkers shows that *intI1* and *intI3* were the most abundant biomarkers. *intI2* was also detected but with low GCN across treatments. Statistical analyses suggest that current wastewater treatment is insufficient at removing MGEs during the fall and winter months. The presence of these MGE biomarkers and clinically relevant ARGs in EFF amplifies the potential risk for the dissemination of antibiotic resistance in wastewater. This includes the transfer of resistance between environmental bacteria as well as pathogenic bacteria such as those in the ESKAPE group, which were detected throughout the NESTP. This is especially concerning given that the measured integron-integrase genes can contain gene cassettes conferring multidrug resistance [74]. Further analysis of integron-integrase GCN differences across more seasons will be required to determine the extent of integron-integrase variation within the NESTP.

This is the first study exploring the microbial composition and resistome of the NESTP, the largest WWTP in Manitoba, using shotgun metagenomics. The results of this study establish a baseline for future studies examining the effect of modifying operational parameters on microbial diversity as well as antibiotic resistance in the NESTP as it continues to be upgraded. This study also contributes to our understanding of the effects of colder climates on the resistome and community composition in a full-scale WWTP.

## Supplementary Information

**Additional file 1**. Supplementary tables and figures

**Additional file 2**. NESTP metadata, MG-RAST taxonomical classification datasets, and real-time PCR datasets

**Additional file 3**. Curated CARD datasets

## Data Availability

The datasets supporting the conclusions of this article are included within the article (and its additional files).

## Abbreviations

ARG: Antibiotic resistance gene
BOD: Biological oxygen demand
CARD: Comprehensive Antibiotic Resistance Database
COD: Chemical oxygen demand
EFF: Effluent
ESKAPE: *Enterococcus faecium, Staphylococcus aureus, Klebsiella pneumoniae, Acinetobacter baumannii, Pseudomonas aeruginosa,* And *Enterobacter* spp
GCN: Gene copy number
MGE: Mobile genetic element
MG-RAST: Metagenomic Rapid Annotation using Subsystem Technology
NESTP: North End Sewage Treatment Plant (Winnipeg, MB, Canada)
PCA: Principal component analysis
PCoA: Principal coordinate analysis
QAC: Quaternary ammonium compounds
RS: Raw sewage or influent
RAS: Returned activated sludge
SC: Dewatered sludge or sludge cake
T1: October 22, 2019 sampling event
T2: November 28, 2019 sampling event
T3: December 18, 2019 sampling event
T4: February 6, 2020 sampling event
TN: Total nitrogen
TP: Total phosphorus
TOC: Total organic carbon
TSS: Total suspended solids
WWTP: Wastewater treatment plant
