# Supplementary material for: Metagenomic community composition and resistome analysis in a full-scale cold climate wastewater treatment plant
Source: Environ Microbiome. 2022 Jan 15;17:3. doi: 10.1186/s40793-022-00398-1 (PMC8760730; doi:10.1186/s40793-022-00398-1)
Supplement: Supplementary file 1 — Additional file 1. Supplementary tables and figures [file 40793_2022_398_MOESM1_ESM.docx]

**Supplemental Tables**

**Table S1.** Metagenomic shotgun sequencing details.

| Treatment | Date sampled | Sample Type | File Size (Gb)* | Total Reads* | Contig Length Range (bp) | MG-RAST ID | SRA Accession |
| --- | --- | --- | --- | --- | --- | --- | --- |
| Raw Sewage (RS) | October 22, 2019 | Bacteria | 0.71 | 1495081 | 151-108304 | mgm4916805.3 | SRR16214424 |
|  | November 28, 2019 | Bacteria | 0.68 | 1430320 | 151-138998 | mgm4916782.3 | SRR16214422 |
|  | December 18, 2019 | Bacteria | 0.91 | 1904436 | 151-185760 | mgm4916801.3 | SRR16214421 |
|  | February 6, 2020 | Bacteria | 0.86 | 1808603 | 151-139377 | mgm4916787.3 | SRR16214420 |
|  | October 22, 2019 | Phage | 1.30 | 2602558 | 151-120380 | mgm4916781.3 | SRR16214419 |
|  | November 28, 2019 | Phage | 1.60 | 3332632 | 151-100322 | mgm4916783.3 | SRR16214418 |
|  | December 18, 2019 | Phage | 1.50 | 3141003 | 151-73130 | mgm4916798.3 | SRR16214417 |
|  | February 6, 2020 | Phage | 0.80 | 1657329 | 151-94453 | mgm4916814.3 | SRR16214416 |
| Returned Activated Sludge (RAS) | October 22, 2019 | Bacteria | 1.20 | 2496292 | 151-147215 | mgm4916793.3 | SRR16214432 |
|  | November 28, 2019 | Bacteria | 1.40 | 2984571 | 151-141803 | mgm4916804.3 | SRR16214431 |
|  | December 18, 2019 | Bacteria | 0.89 | 1861066 | 151-102513 | mgm4916786.3 | SRR16214430 |
|  | February 6, 2020 | Bacteria | 1.20 | 2548769 | 151-103306 | mgm4916809.3 | SRR16214429 |
|  | October 22, 2019 | Phage | 1.40 | 2785175 | 151-190578 | mgm4916803.3 | SRR16214428 |
|  | November 28, 2019 | Phage | 0.63 | 1329879 | 151-91442 | mgm4916808.3 | SRR16214427 |
|  | December 18, 2019 | Phage | 0.49 | 1022720 | 151-66976 | mgm4916795.3 | SRR16214426 |
|  | February 6, 2020 | Phage | 1.50 | 3081713 | 151-114501 | mgm4916802.3 | SRR16214425 |
| Effluent (EFF) | October 22, 2019 | Bacteria | 0.99 | 2110661 | 151-34755 | mgm4916794.3 | SRR16214435 |
|  | November 28, 2019 | Bacteria | 0.61 | 1277227 | 151-197648 | mgm4916806.3 | SRR16214434 |
|  | December 18, 2019 | Bacteria | 0.87 | 1894603 | 151-182074 | mgm4916807.3 | SRR16214423 |
|  | February 6, 2020 | Bacteria | 0.85 | 1806721 | 151-132128 | mgm4916791.3 | SRR16214412 |
|  | October 22, 2019 | Phage | 0.80 | 1660030 | 151-76963 | mgm4916796.3 | SRR16214404 |
|  | November 28, 2019 | Phage | 1.20 | 2509560 | 151-70152 | mgm4916815.3 | SRR16214403 |
|  | December 18, 2019 | Phage | 1.80 | 3546951 | 151-70224 | mgm4916788.3 | SRR16214402 |
|  | February 6, 2020 | Phage | 0.66 | 1308426 | 151-86350 | mgm4916797.3 | SRR16214401 |
| Dewatered Sludge (SC) | December 18, 2019 | Bacteria | 0.79 | 1663544 | 151-44761 | mgm4916792.3 | SRR16214415 |
|  | February 6, 2020 | Bacteria | 0.96 | 2000191 | 151-40611 | mgm4916800.3 | SRR16214414 |
|  | December 18, 2019 | Phage | 1.40 | 2993037 | 151-47652 | mgm4916799.3 | SRR16214413 |
|  | February 6, 2020 | Phage | 0.05 | 88061 | 151-18841 | mgm4916785.3 | SRR16214411 |
| Negative Control (NEG) | October 22, 2019 | Bacteria | 6.30 Mb | 12729 | 151-1107 | mgm4916813.3 | SRR16214409 |
|  | November 28, 2019 | Bacteria | 5.30 Mb | 10343 | 157-923 | mgm4916784.3 | SRR16214408 |
|  | December 18, 2019 | Bacteria | 1.17 Kb | ‡ | 154-671 | ‡ | SRR16214407 |
|  | February 6, 2020 | Bacteria | 1.58 Kb | ‡ | 169-691 | ‡ | SRR16214406 |
|  | † | Phage | 9.20 Mb | 18278 |  | mgm4916790.3 | SRR16214405 |
| Mock Community | - | Bacteria | 2.00 | 4290164 | 199-1942987 | mgm4916810.3 | SRR16214400 |
|  | - | Bacteria | 0.87 | 1848661 | 183-613621 | mgm4916812.3 | SRR16214399 |
|  | - | Phage | 0.39 | 816011 | 151-194642 | mgm4916789.3 | SRR16214433 |

* After paired-end merge and filtering.

^†^ Negative controls combined across the four sampling periods.

^‡^ File too small to upload to MG-RAST.

**Table S2.** R packages used for network analysis and visualization.

| **Package name** | **Version** | **Reference** |
| --- | --- | --- |
| *devtools* | 2.4.2 | [1] |
| *readxl* | 1.3.1 | [2] |
| *report* | 0.3.5 | [3] |
| *rstudioapi* | 0.13 | [4] |
| *Hmisc* | 4.5-0 | [5] |
| *reshape2* | 1.4.4 | [6] |
| *tidyverse* | 1.3.1 | [7] |

**Supplemental Figures**

**
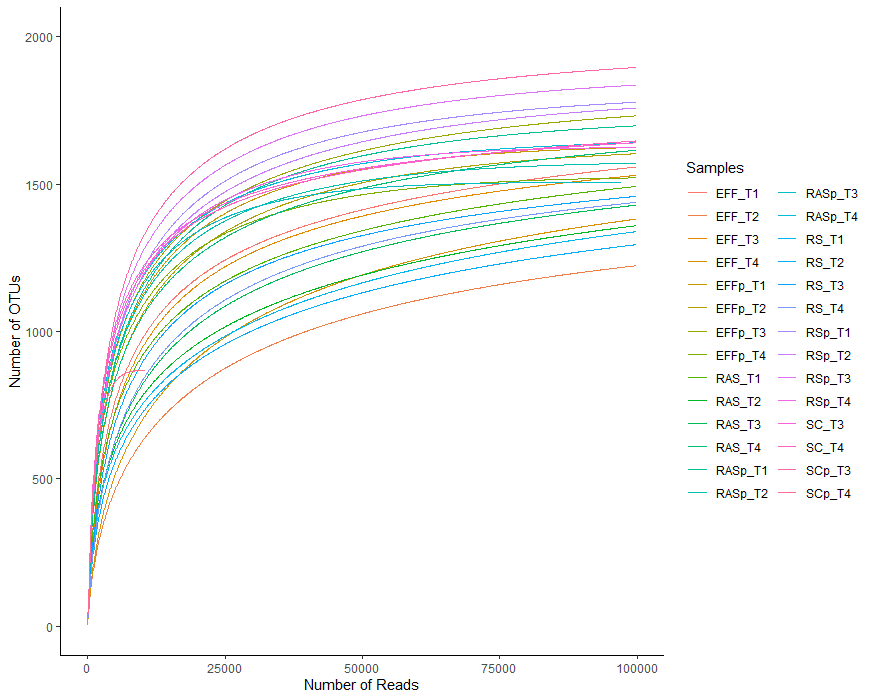
**

**Figure S1.** Rarefaction curve of each metagenomic shotgun sequencing sample. Species-level count data was obtained from MG-RAST with singletons and doubletons removed. Effluent (EFF), returned activated sludge (RAS), raw sewage/influent (RS), dewatered sludge (SC), negative control (NEG), October (T1), November (T2), December (T3), February (T4), notation of “p” indicates phage sample.


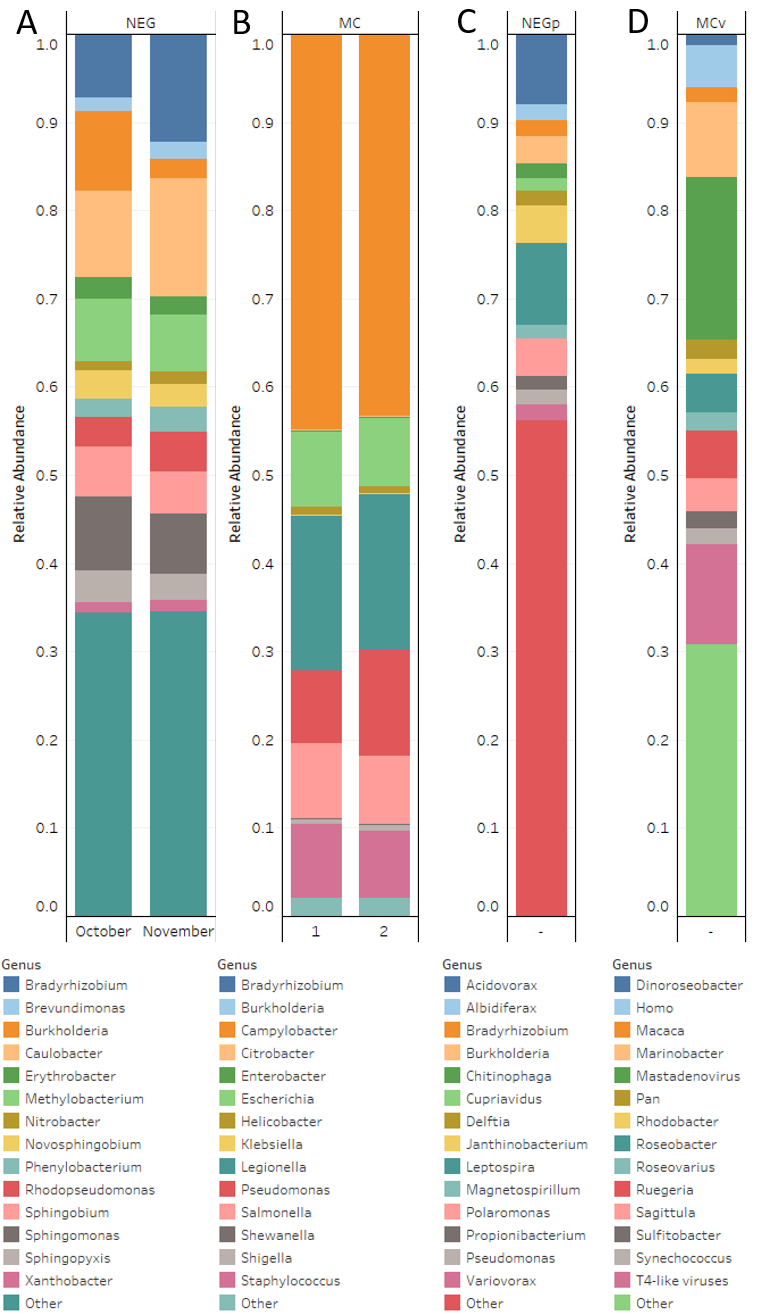


**Figure S2**. Composition of bacteria and phage at the genus level in the control samples. (A) Bacterial fraction negative control relative abundance. (B) Bacterial mock community relative abundance. (C) Viral fraction negative control relative abundance. (D) Viral mock community relative abundance. Mock community (MC), negative control (NEG), viral mock community (MCv), viral negative control (NEGp).


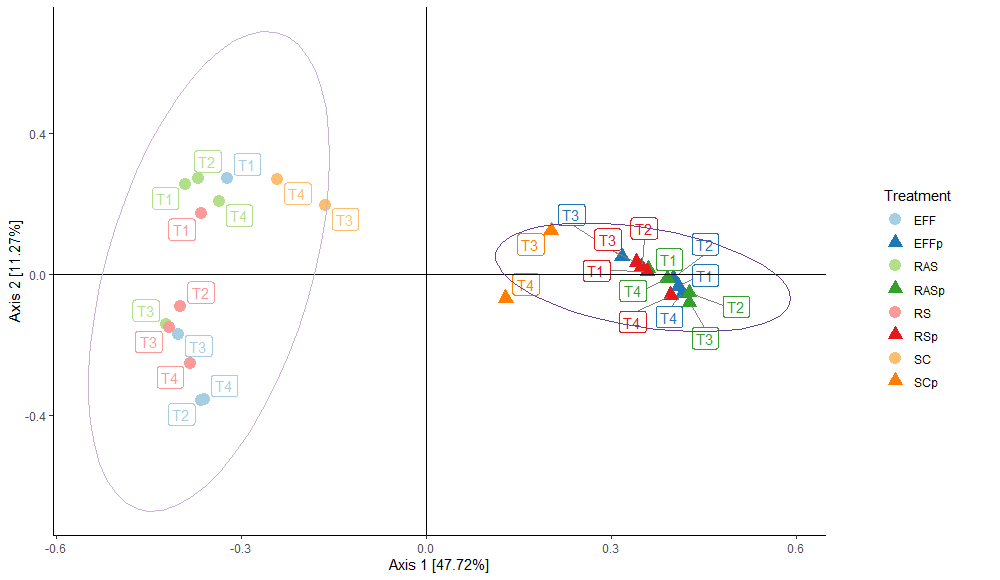


**Figure S3.** Comparison of unstratified bacteria and phage samples by principal coordinate analysis across treatments. Bray-Curtis distance matrix was used with the species taxonomic data from MG-RAST. Principal components 1 and 2 explain 58.99% of the variation. Effluent (EFF), returned activated sludge (RAS), raw sewage/influent (RS), dewatered sludge (SC), negative control (NEG), October (T1), November (T2), December (T3), February (T4), notation of “p” indicates phage sample.


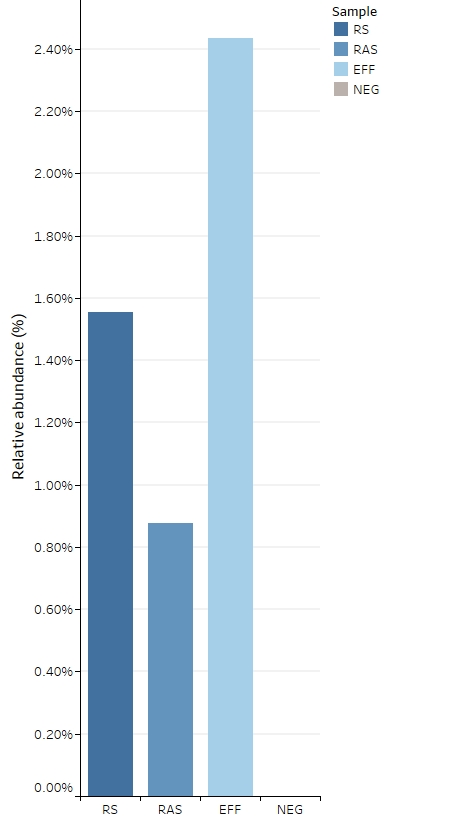


**Figure S4.** Ratio of *uidA* to 16S rRNA gene over each treatment process expressed as percentage. The average of each month was expressed for the four treatments. Effluent (EFF), returned activated sludge (RAS), raw sewage/influent (RS), negative control (NEG).

**
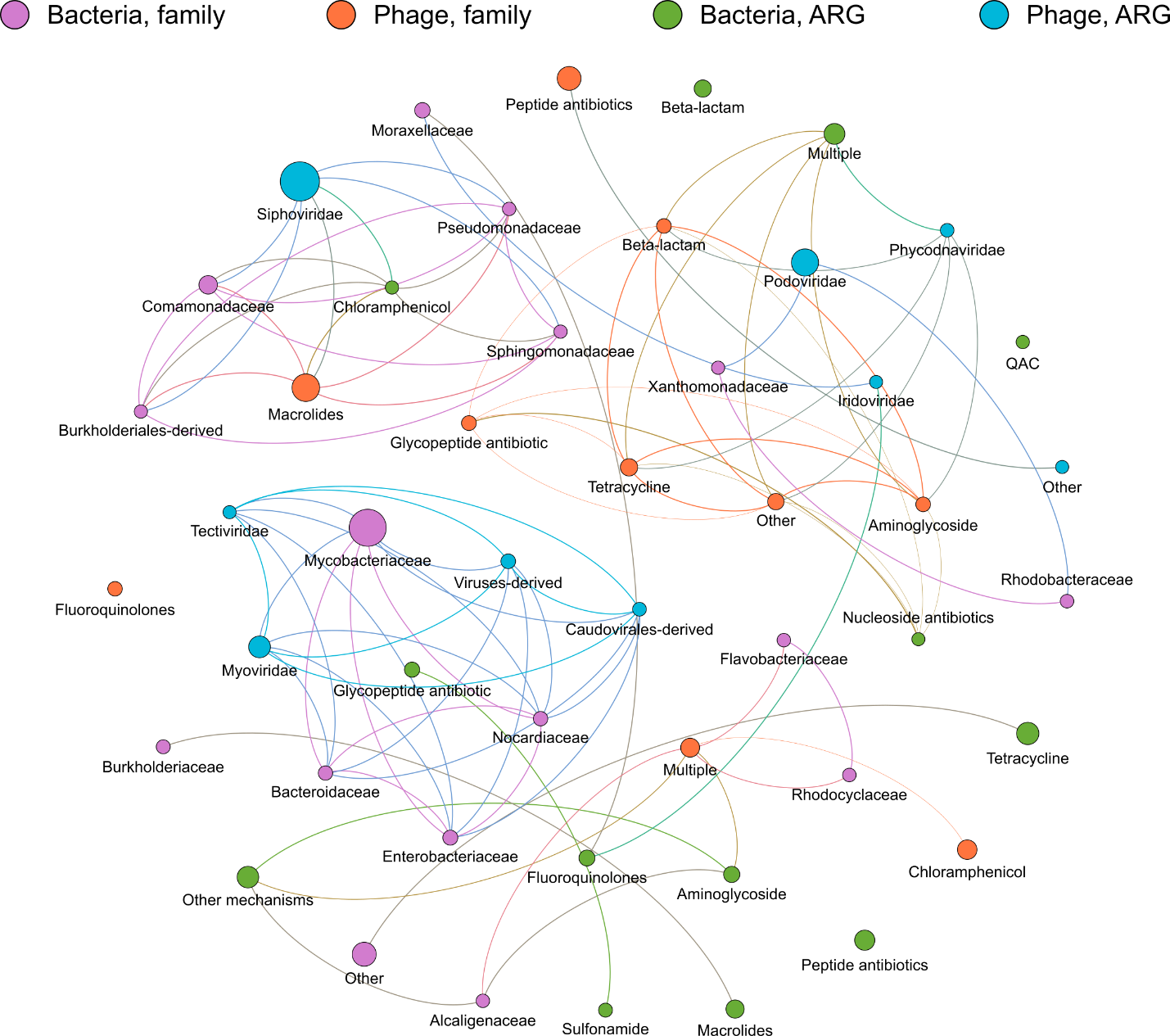
**

**Figure S5**. Network analysis of co-occurrence patterns among ARGs and microbial taxa in RS samples. Node sizes correspond to relative abundances. Antibiotic resistance gene (ARG), raw sewage (RS).


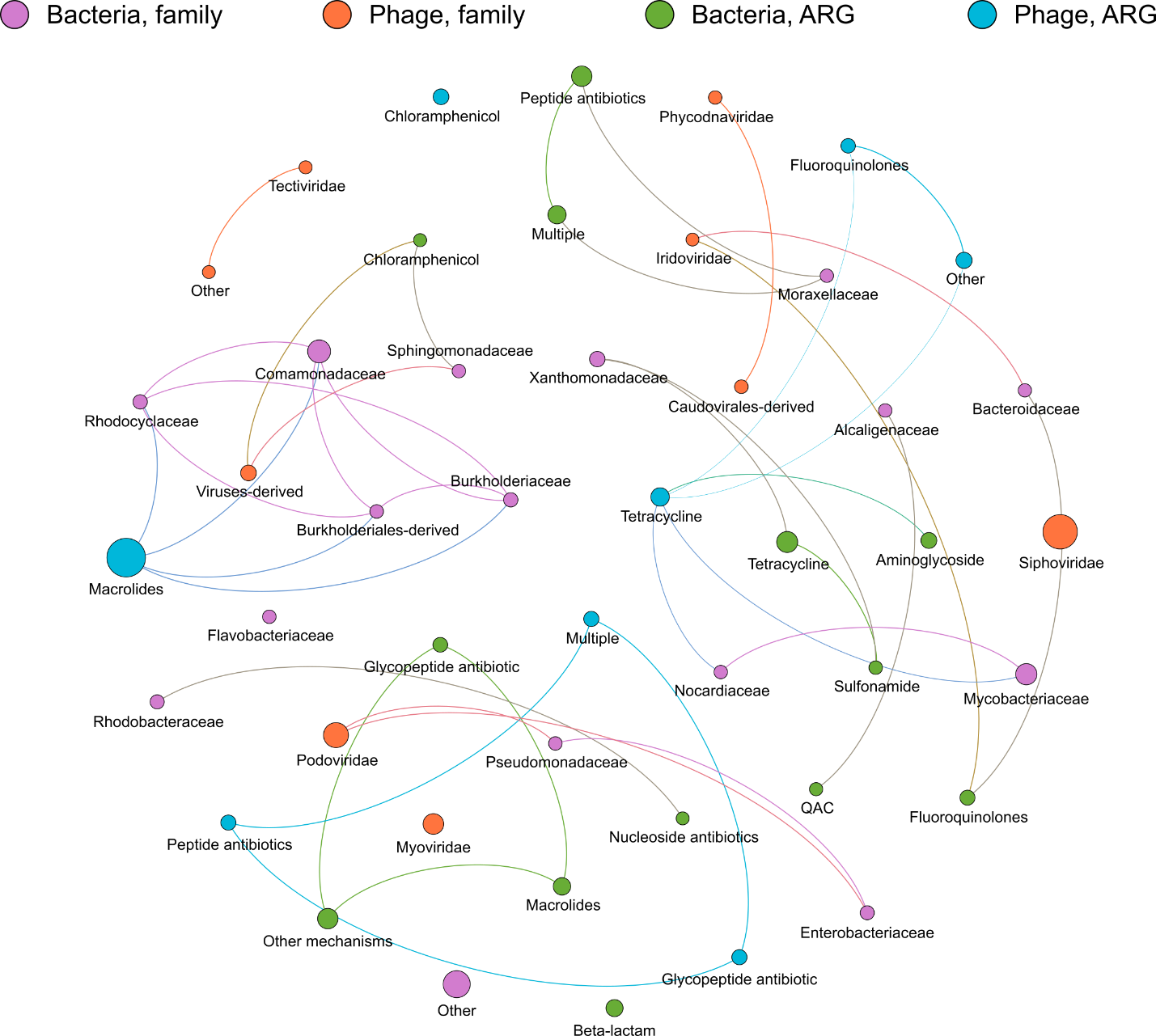


**Figure S6**. Network analysis of co-occurrence patterns among ARGs and microbial taxa in RAS samples. Node sizes correspond to relative abundances. Antibiotic resistance gene (ARG), returned activated sludge (RAS).


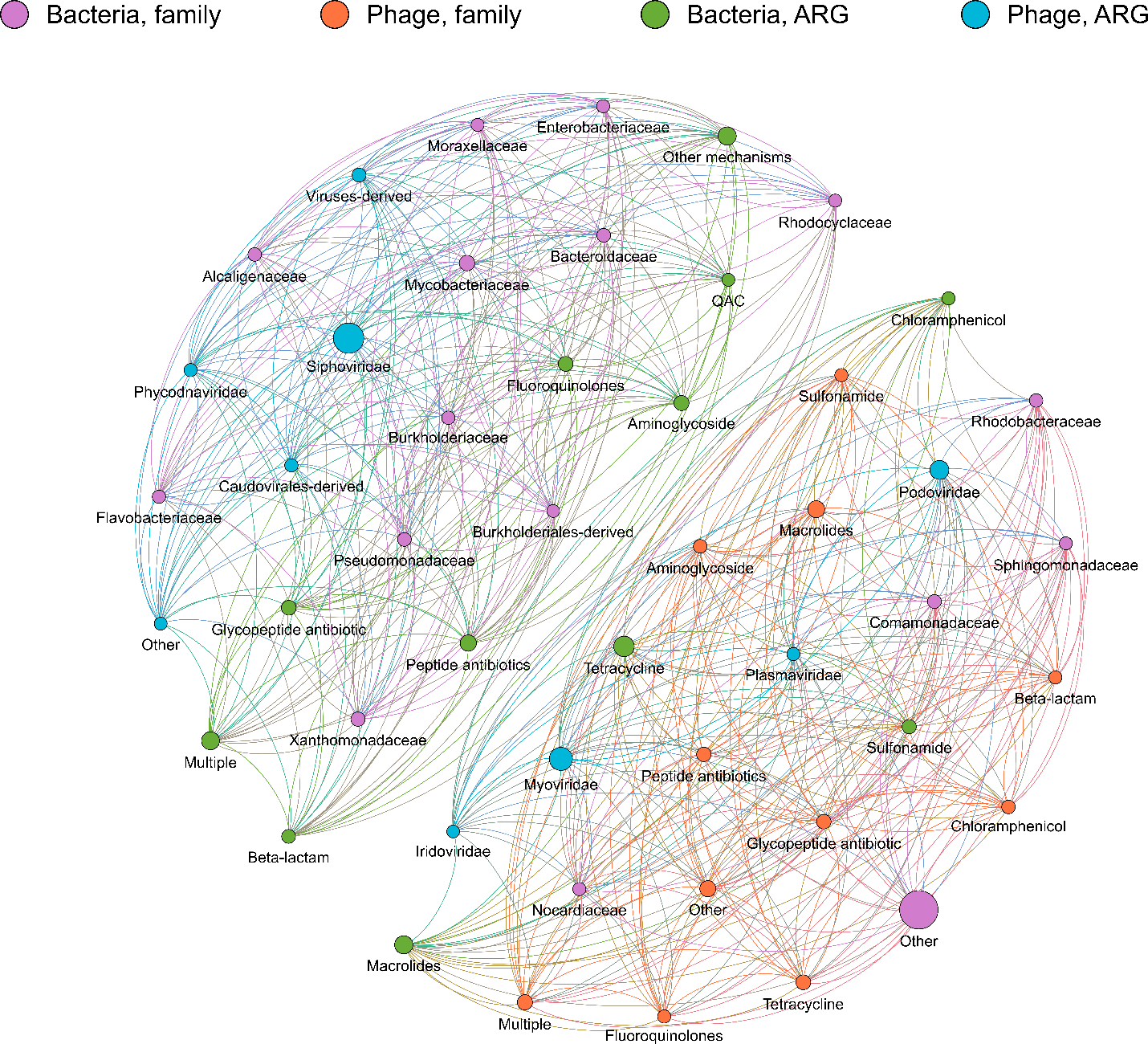


**Figure S7**. Network analysis of co-occurrence patterns among ARGs and microbial taxa in SC samples. Node sizes correspond to relative abundances. Antibiotic resistance gene (ARG), dewatered sludge (SC).

**Supplemental References:**

1. Wickham H, Hester J, Chang W. devtools: Tools to Make Developing R Packages Easier. 2021. https://cran.r-project.org/package=devtools.

2. Wickham H, Bryan J. readxl: Read Excel Files. 2019. https://cran.r-project.org/package=readxl.

3. Makowski D, Ben-Shachar M., Patil I, Lüdecke D. Automated Reporting of Results and Statistical Models. 2020. https://github.com/easystats/report.

4. Ushey K, Allaire J, Wickham H, Ritchie G. rstudioapi: Safely Access the RStudio API Access. 2020. https://cran.r-project.org/package=rstudioapi.

5. Harrel Jr FE. Hmisc: Harrell Miscellaneous. 2021. https://cran.r-project.org/package=Hmisc.

6. Wickham H. reshape2: Flexibly Reshape Data: A Reboot of the Reshape Package. 2020. https://cran.r-project.org/package=reshape2.

7. Wickham H, Averick M, Bryan J, Chang W, McGowan L, François R, et al. Welcome to the Tidyverse. J Open Source Softw. 2019;4:1686.
